# A systematic review of the accessibility, acceptability, safety, efficiency, clinical effectiveness, and cost-effectiveness of private cataract and orthopedic surgery clinics

**DOI:** 10.1017/S0266462323000120

**Published:** 2023-08-01

**Authors:** Ilke Akpinar, Erin Kirwin, Lisa Tjosvold, Dagmara Chojecki, Jeff Round

**Affiliations:** 1 Institute of Health Economics, Edmonton, AB, Canada; 2 Department of Pediatrics, Faculty of Medicine, University of Alberta, Edmonton, AB, Canada

**Keywords:** private surgical facilities, cataract, orthopedic surgery, systematic review

## Abstract

**Objectives:**

Many publicly funded health systems use a mix of privately and publicly operated providers of care to deliver elective surgical services. The aim of this systematic review was to assess the role of privately operated but publicly funded provision of surgical services for adult patients who had cataract or orthopedic surgery within publicly funded health systems in high-income countries.

**Methods:**

Electronic databases (Ovid MEDLINE, OVID Embase, and EBSCO EconLit) were searched on 26 March 2021, and gray literature sources were searched on 6 April 2021. Two reviewers independently applied inclusion and exclusion criteria to identify studies, and extracted data. The outcomes evaluated include accessibility, acceptability, safety, clinical effectiveness, efficiency, and cost/cost-effectiveness.

**Results:**

Twenty-nine primary studies met the inclusion criteria and were synthesized narratively. We found mixed results across each of our reported outcomes. Wait times were shorter for patients treated in private facilities. There was evidence that some private facilities cherry-pick or cream-skim by selecting less complex patients, which increases the postoperative length of stay and costs for public facilities, restricts access to private facilities for certain groups of patients, and increases inequality within the health system. Seven studies found improved safety outcomes in private facilities, noting that private patients had a lower preoperative risk of complications. Only two studies reported cost and cost-effectiveness outcomes. One costing study concluded that private facilities’ costs were lower than those of public facilities, and a cost–utility study showed that private contracting to reduce public waiting times for joint replacement was cost-effective.

**Conclusions:**

Limited evidence exists that private-sector contracts address existing healthcare delivery problems. Value for money also remains to be evaluated properly.

## Background

Over the past two decades, governments have tried to reduce costs in health care while improving access and reducing wait times. Although health systems and funding types vary between countries, a common challenge is dealing with increasing demand and healthcare expenses while providing efficient and high-quality care (1). Quality improvement interventions, which redesign access to services, make changes to market structures, and create a competitive environment, are advocated for in many countries, including Canada, the United Kingdom (UK), and Australia (2). These improvements have been particularly important in the context of elective surgeries (1–3). Although the use of private providers within the publicly funded health system has always been controversial, delivery-side market-oriented reforms in health care have been adopted widely. Under typical reforms, universality of coverage through taxation remains, but a competitive environment has been introduced on the supply side. Independent Sector Treatment Centres (ISTC) were introduced by the UK government in the 2000s with the primary aim of providing high-volume elective surgeries, such as cataract surgeries, hip and knee replacements (4;5).

With increasing pressures on public health systems internationally, there is a need to develop evidence based on the effects of private elective surgical provision within public health systems. Many studies have been produced to investigate the various effects of private elective surgical provision (6–19), and systematic reviews (20) and overviews (21) have provided evidence on public health outcomes compared to private provision of health services. Our focus was cataract and orthopedic surgeries as they persistently have long waitlists in many jurisdictions (22;23). As far back as 2004, hip and knee replacement and cataract surgery were listed as priority procedures in Canada, and Canadian Institute for Health Information (CIHI) was mandated to collect wait time information. According to a CIHI report, almost half of the Canadians who received a hip, knee replacement, or cataract surgery waited longer than recommended (22). Cataract lens insertion, and knee and hip replacement are the top three high-volume implantable medical device procedures in Canada (24). The CIHI 2020 report states that cataract lens insertion is the most common surgical procedure with 413, 202 procedures performed in 2018–19, followed by knee replacement and hip replacement with 75, 220 and 65, 645 procedures, respectively (24). To the best of our knowledge, no systematic reviews focus on the role of private provision of these elective surgeries in publicly funded health systems.

This systematic review aimed to identify differences in outcomes between public- and private-sector provision of cataract and orthopedic surgical procedures within publicly funded health systems. Our goal is to understand the benefits and drawbacks of both public and private provision of care, and to help inform policy makers of the trade-offs associated with different policy options.

## Materials and methods

The systematic review was carried out using predefined protocol. Following inclusion and exclusion criteria and using data extraction form, each step was conducted by two reviewers independently.

### Data sources and search strategy

The literature search was designed to identify studies that investigated the accessibility, acceptability, safety, clinical effectiveness, efficiency, and cost-effectiveness of private surgical facilities. Relevant bibliographic databases (Ovid MEDLINE, OVID Embase, and EBSCO EconLit) and gray literature sources (Google Advanced, The King’s Fund, OECD, European Observatory, Commonwealth Fund, Conference Board of Canada, Fraser Institute, INAHTA, nd CADTH) were searched by an information specialist (DC) between 14 and 21 May 2019. The first search update was performed by a second information specialist (LT) between 26 March and 6 April 2021 on the same sources, and a second search update was performed (DC) between 3 and 9 October 2022. Relevant studies published from January 2000 onwards were identified using a combination of controlled vocabulary (MeSH and EMTREE terms) and keywords relating to private non-hospital surgical facilities and the contracting out of services to them by the public health sector. Full details of the search strategy can be found in Additional file 1.

### Inclusion and exclusion criteria

Studies were included if they met the following criteria:

- Study design: Primary studies and systematic reviews
- Population: Adult patients who had a cataract or orthopedic surgery
- Setting: Publicly funded health systems non-hospital/hospital private surgical facilities and surgical facilities (both private and public hospital settings) operated by public sector health providers (e.g., national or regional health authorities)
- Intervention: cataract and orthopedic surgeries operated in non-hospital/ hospital public facilities
- Comparator: cataract and orthopedic surgeries operated in non-hospital/hospital private facilities
- Outcome measures: Accessibility (waiting times, availability of health professionals or centers), acceptability (public/patient perceptions), safety/quality of care (readmission and complication rates), clinical effectiveness (need for revision), efficiency and cost/cost–benefit/cost-effectiveness of private/public surgical facilities

Studies that did not report data on any of the pre-defined outcomes were excluded.

### Study selection

Titles and abstracts were screened by two reviewers (IA and EK) and full-texts of the potentially relevant articles were retrieved. Disagreements over eligibility and study quality were resolved by discussion. A third (JR) reviewer helped resolve uncertainty when needed.

To be eligible for inclusion, a study must have (i) evaluated the impact of private surgical facilities within publicly funded health systems, (ii) included at least one of the outcomes described above, and (iii) the provision of elective cataract and orthopedic services only.

### Data extraction

The following data were extracted from studies: author(s) name, country of the study, year of publication, title, objective, surgery type, setting, population, outcome measures, results, and conclusion. Two reviewers (IA and EK) extracted data in duplicate from the selected primary studies. We extracted data separately for cataract and orthopedic surgeries. Systematic reviews were not included for analysis if they did not adequately address our scope on cataract and orthopedic surgery. Owing to the heterogeneity of study designs we did not undertake formal evidence synthesis, and instead report the results of each study.

### Quality assessment

Studies were assessed by author AI against relevant JBI quality appraisal checklists (25–29) and the Consolidated Health Economic Evaluation Reporting Standards (CHEERS) 2022 (30) reporting checklist as appropriate. We report the proportion of checklist items that were met for each study (Tables 1–5, Additional File 1).

**Table 1.** Studies evaluating accessibility, acceptability, and safety

Accessibility						
Surgery type	Study	Proportion of checklist items met	Country	Population	Outcome measure	Results
Cataract	Kruse et al. 2019	0.82	Netherlands	50 000 patients who received cataract care (including academic and tertiary hospitals) from 2013 to 2015. Average age: 72.26 ± 9.77 (private) vs. 73 ± 10.1 (public)	surgery volume	The average number of surgeries is higher within private facilities, with, on average, 0.91 cataract operations per care pathway, whereas general hospitals have an average of 0.84.
	Pager and McCluskey 2004	0.80	Australia	Forty-two public patients and 39 private patients in Sydney, Australia between April and June 2002. Average age: 71.7 ± 9.6 (private) vs. 72.9 ± 9.4 (public)	wait time for surgery	Mean surgery wait times for patients at public hospitals were nine times longer (38.2 vs. 4.4 weeks, *p* < .001) than the private center patients.
	Solborg et al. 2015	0.73	Denmark	Cataract Surgery patients in Denmark between 2004 and 2012. 243 856 patients (411 140 cataract operations).	wait time for second eye surgery	The median time interval between the first and second eye cataract surgery was 7 days shorter for patients who had cataract surgery in private hospitals compared to patients who had cataract surgery in public hospitals (95% CI:6.65–7.35, p < .001).
Orthopedic	Andersen and Jakobsen 2011	0.80	Denmark	Hip Arthroplasty patients. 8149 patients from 36 public and 20 private clinics in Denmark for fiscal years 2007 to 2008.	wait times	Private clinics have shorter waiting times than public clinics both for preliminary examinations (0.41 vs. 7.43 weeks) and actual operations (0.82 vs. 6.94 weeks).
	Fitzpatrick et al. 2004	0.80	UK	Patients of recruited physicians in need of hip replacement surgery in England from September 1996 and October 1997. 13 343 surgeries were performed in 143 public hospital and 390 private facilities. Median age: 68.5	wait times	Publicly funded patients were more likely (OR = 5.28, 95% CI 4.22–6.59) to report a wait time greater than 3 months for an outpatient appointment and more likely (OR = 12.80, 95% CI 9.81–16.68) report a wait time longer than 6 months for total hip replacement (THR) than the privately funded ones.
	Kelly and Stoye 2016	0.82	UK	Elective hip replacement patients between April 2002 and March 2011 analyzed for 6 781 aggregated geographic areas.	wait times	Waiting times did not depend on the nearest Independent Sector Treatment Center (ISTC); however, the introduction of the private-sector providers reduced waiting times at National Health Service (NHS) hospitals without any effect on surgical volumes.
	Kelly and Stoye 2020	0.82	UK	Elective hip replacement patients (615,281 patients) between April 2002 and March 2013.	wait times	In 2012–13, median wait times were significantly lower at private hospitals than in public hospitals at 62.8(101.7) days and 91.5(59.2) days, respectively.
	Kirkwood and Pollock 2017	1.00	UK	Patients receiving publicly funded primary hip arthroplasty (105 872 elective) in Scotland between April 1993 and March 2013.	treatment inequalities	Increased use of private-sector provision was associated with a significant decrease in direct NHS provision in 2008/09 (*p* < .01) and with widening inequalities by age or socio-economic deprivation.
	Koehoorn et al. 2011	0.82	Canada	Workers with accepted workers compensation claim for meniscal injury. 1380 surgeries from 2001 to 2005 in British Columbia, Canada. Average age: 46.1 (private) vs. 44.7 (public)	wait time for surgery	Wait time for surgery (median days, IQR) 22 (12,38) public hospital vs. 24 (13,39) private hospitals.
	Moscone et al. 2019	0.73	Italy	Acute myocardial infarction, stroke, hip and knee replacement patients admitted to 189 Italian hospitals located in the Lombardy region between 2012 and 2014. Age: 68.25 (hip replacement), 70.12 (knee replacement)	wait time for surgery	Ordinary least square regression analysis results indicated that hip and knee surgery wait times are shorter in private hospitals by approximately 25–35%.
Both	Tulp et al. 2020	0.75	Netherlands	Patients who had anterior cruciate ligament surgery, cataract surgery, total hip replacement, total knee replacement, and carpal tunnel syndrome surgery in Dutch hospitals and ISTCs in 2017.	surgery volume	Cataract (1855.22 ± 965.50 vs. 1180.81 ± 640.65), hip (379.51 ± 184.63 vs. 127.92 ± 130.68), and knee surgeries (315.00 ± 149.90 vs. 163.07 ± 182.84) were performed more frequently in public hospitals.

Acceptability						
Surgery Type	Study		Country		Outcome measure	Results
Cataract	Kruse et al. 2019	0.82	Netherlands	50 000 patients who received cataract care (including academic and tertiary hospitals) from 2013 to 2015. Average age: 72.26 ± 9.77 (private) vs. 73 ± 10.1 (public)	patient satisfaction level	Using the Net Promoter Score and PROM to measure patient satisfaction and identified significantly higher scores among private clinic patients compared with public hospital patients. p < .01
	Pager and McCluskey 2004	0.80	Australia	Cataract surgery patients. Forty-two public patients and 39 private patients in Sydney, Australia between April and June 2002. Average age: 71.7 ± 9.6 (private) vs. 72.9 ± 9.4 (public)	quality of information, level of satisfaction	Public sector patients were less satisfied overall than private-sector patients, although the level of satisfaction between groups was similar.2.1(0.6) vs. 1.7(0.4) p = 0.002
Orthopedic	Adie et al. 2012	0.82	Australia	Scheduled hip or knee surgery patients in Sydney, Australia from April 2007 to December 2008. (331 total, 184 public, 147 private; 215 knees, 116 hips). Average age: 68 ± 12.3 (private) vs. 68 ± 9.8 (public)	patient satisfaction level	Almost 90% satisfaction rates in public and private patients at six and 12 months after surgery. After adjusting for the effect of patient expectation (expectations met in 76% of private centers and 64% of public centers at 12 months), private patients were less likely to be satisfied due to their higher expectations (adjusted odds ratio [aOR] for satisfaction at 12 months: 0.16 private vs. public).
	Andersen and Jakobsen 2011	0.80	Denmark	Hip Arthroplasty patients. 8 149 patients from 36 public and 20 private clinics in Denmark for fiscal years 2007 to 2008.	patient satisfaction level	Patient satisfaction levels are higher for private clinics than public clinics.
	Naylor et al. 2016	1	Australia	Hip and knee replacement patients in Australia from 2014–2015. Telephone survey with 457 survey responders (210 private).	patient satisfaction level, hospitality and frequency of surgeon visitation	Public sector consumers were significantly more satisfied than private-sector consumers (OR 1.56 95% CI 1.16–2.10, p < .01), but there was no significant difference (p = 0.85) for likeliness to recommend between the sectors. The only care domains where the private out-performed the public sector were hospitality (46.7 vs. 35.6% p < .01) and frequency of surgeon visitation (76.4 vs. 65.8%, p = 0.03).
Both	Browne et al. 2008		UK	769 patients (inguinal hernia, varicose vein, and cataract) treated in six private and 1895 treated in 20 public facilities in England during 2006–07. Age: 73.7 ± 10.6 (cataract public), 74.6 ± 10.0 (cataract private), 66.2 ± 14.6 (hip public), 66.8 ± 14.1 (hip private), 66.2 ± 16.5 (knee public), 66.7 ± 12.2(knee private).	patient-reported health status	Most patients described the result of their operation as a success (excellent, very good or good) both in ISTCs and NHS facilities: cataract surgery 97% v 91%; hip replacement 98% v 92%; and knee replacement 85% v 87%.
	Perotin et al. 2013		UK	Patients following discharge from public (NHS) and private (ISTC) facilities from 2007 to 2008 in the UK, who are selected to complete a postal questionnaire. 21 680 cases in NHS hospitals and 16 767 cases in ISTCs (linked to 2007 NHS trust Inpatient Survey and the 2007 and 2008 ISTC Inpatient and Day Case quarterly patient surveys)	quality of information, care, privacy, dignity, hospitality, and procedural delays	Public sector hospitals provided better information [0.0363(1.2752)] and more choice, whereas private-sector facilities offered a more comfortable, friendly, and clean environment [−0.1422(0.76665)].

Safety						
Surgery type	Study		Country		Outcome measure	Results
Cataract	Li et al. 2004		Australia	All patients who underwent cataract surgery (117 083 procedures), along with those in whom postoperative endophthalmitis subsequently developed from 1980 to 2000 in Western Australia.	postoperative infection	Procedures performed in private hospitals had a significantly higher risk of postoperative infection than did those performed in public hospitals (OR 2.38; 95% CI 1.32–4.27).
	Solborg et al. 2015		Denmark	Cataract Surgery patients in Denmark between 2004 and 2012. 243 856 patients (411 140 cataract operations).	mortality rate	The overall mortality in patients who had regular cataract surgery in public hospitals was 62% (IRR: 1.62, 1.59 < 95% CI: −1.66, p < .001, Poisson regression) higher compared to patients who had cataract surgery in private hospitals/clinics.
	Solborg et al. 2013		Denmark	PE cases in cataract surgery patients identified via the National Public Registry in Denmark between 2002 and 2010 (107 701 registered operations).	postoperative infection	The relative postoperative endophthalmitis risk for private clinics compared with the eye departments in public hospitals was 8.58 (95% CI = 5.09–14.47; p < 0.0005)
Orthopedic	Andersen and Jakobsen 2011		Denmark	Hip Arthroplasty patients. 8 149 patients from 36 public and 20 private clinics in Denmark for fiscal years 2007 to 2008.	complication rates, 30-day readmission rates	Percentage of primary hip patients with post-surgery complications (public 2.83 vs. private 0.54). Percentage of primary hip patients acutely readmit within 30 days (public 8.36 vs. private 9.60). Percentage of primary hip patients hospitalized within 3 months due to hip problems (public 3.96 vs. private 5.06).
	Bannister et al. 2010		UK	Hip and knee arthroplasty patients in the UK from October 2003 to March 2005. Patients who had 880 total hip and 874 total knee arthroplasties at a regional orthopedic hospital with 368 total hip and 365 total knee arthroplasties from an NHS and 67 total hip and 86 total knee arthroplasties from a private hospital independent treatment centre.	short term complications (dislocation rates, and wound problems)	Dislocation rates after THR were 6% at private and 1.8% at public hospitals. Major wound problems were 20% at private, 3.8% at public hospitals after hip arthroplasty and 19% at private 1.9% at public after total knee replacement (TKR).
	Chard et al. 2011		UK	Patients undergoing hip or knee replacement (5671 in ISTCs and 14 292 in NHS), inguinal hernia repair (640 and 2023, respectively), or surgery for varicose veins (248 and 1336, respectively) in the UK from June 2008 to September 2009.	complication rates	After adjustment for preoperative differences, patients undergoing joint replacements in NHS providers more often reported complications: OR 1.3 (95% CI 1.1 to 1.5) for hip and 1.4 (1.2 to 1.6) for the knee.
	Holom and Hagen 2017		Norway	All publicly financed patients having primary total hip (37 897 patients) or primary total knee arthroplasty (25 802 patients) at one of the three hospital types from 2009 to 2014 in Norway. Average age: 66.07 ± 10.18 (private) vs. 68.26 ± 11.20 (public)	30-day readmission rates	Private non-profit hospitals had significantly lower rates for 30-day readmission due to complications (0.049 ± 0.217 for private not-for-profit hospitals and 0.080 ± 0.272 for public hospitals).
	Kelly and Stoye 2020		UK	Elective hip replacement patients (615,281 patients) between April 2002 and March 2013	30-day emergency readmission rates	30-day emergency readmission rates following hip replacement were lower at private hospitals: 3.54(18.5) % private vs. 5.61(23.03) % public.
	Moscone et al. 2019		Italy	Acute myocardial infarction, stroke, hip and knee replacement patients admitted to 189 Italian hospitals located in the Lombardy region between 2012 and 2014. Age: 68.25 (hip replacement), 70.12 (knee replacement)	30-day emergency readmission rates	Readmission risk following hip replacement in private facilities was 3.6% higher than in public facilities (p = 0.032).
	Naylor et al. 2016		Australia	Hip and knee replacement patients in Australia from 2014–2015. Telephone survey with 457 survey responders (210 private).	complication rates	Complication rates after TKR 37% public vs. 23% private (p < .01)
Both	Browne et al. 2008		UK	769 patients (inguinal hernia, varicose vein, and cataract) were treated in six private and 1895 treated in 20 public facilities in England during 2006–07. Age: 73.7 ± 10.6 (cataract public), 74.6 ± 10.0 (cataract private), 66.2 ± 14.6 (hip public), 66.8 ± 14.1 (hip private), 66.2 ± 16.5 (knee public), 66.7 ± 12.2(knee private).	postoperative problems	Patients treated in ISTCs were less likely to report postoperative problems than those treated in NHS facilities for cataract surgery (Adjusted Odds Ratio 0.35; 95% CI 0.17–0.70) and knee replacement (0.44; 0.28–0.69).

## Results

### Description of selected studies

**Figure 1.**
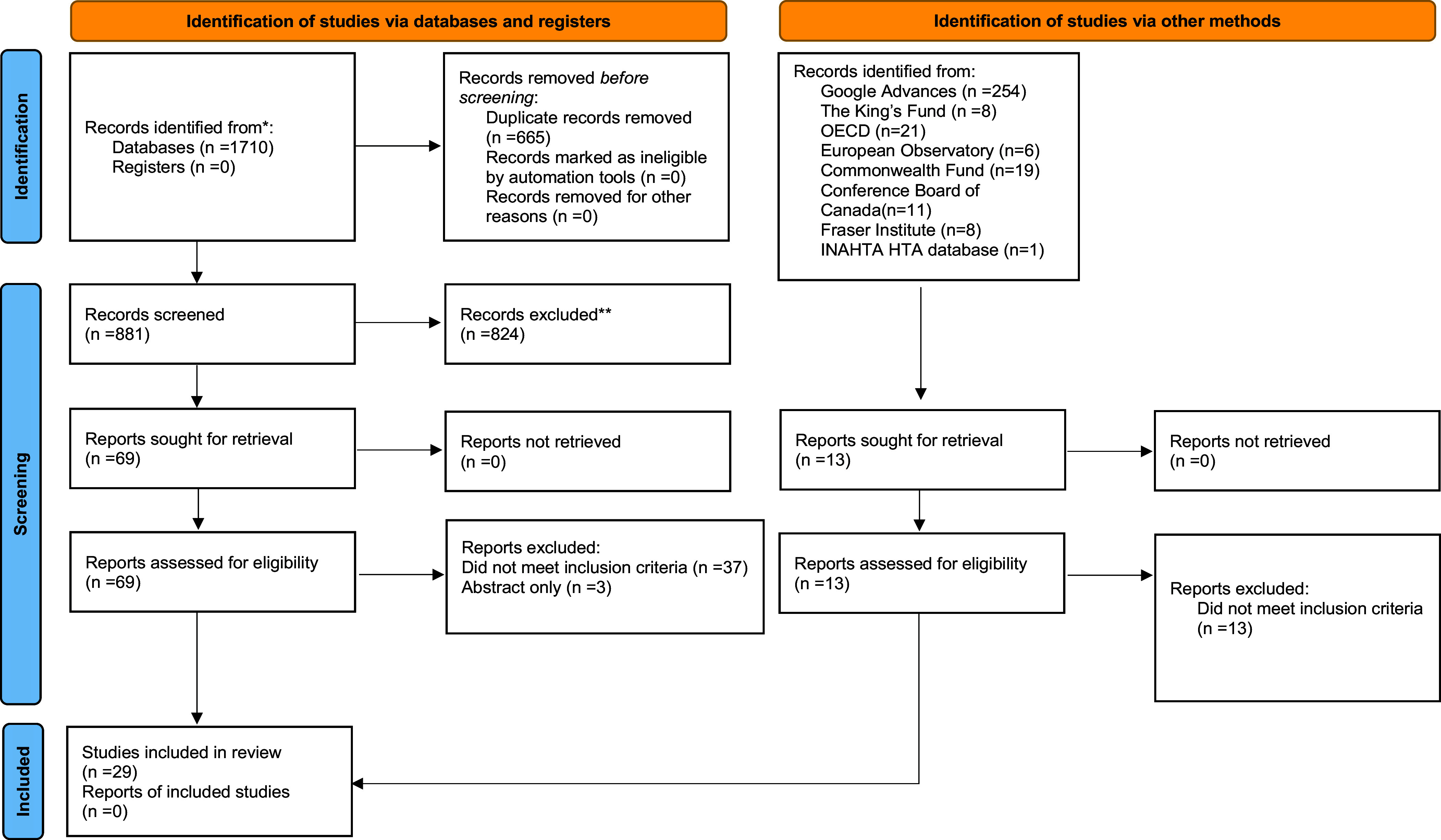
PRISMA 2020 flow diagram. *From:* Page MJ, McKenzie JE, Bossuyt PM, Boutron I, Hoffmann TC, Mulrow CD, et al. The PRISMA 2020 statement: an updated guideline for reporting systematic reviews. BMJ 2021;372:n71. doi: 10.1136/bmj.n71. For more information, visit: http://www.prisma-statement.org/.

Using the PRISMA 2020 (Preferred Reporting Items for Systematic Reviews and Meta-Analyses) flow diagram, Figure 1 indicates the total number of articles through the identification and selection process. The initial and updated main literature search identified 1710 citations (746 citations from MEDLINE, and 964 from EMBASE and EconLit electronic databases). A total of 328 gray literature results were identified. After duplicates were removed, 1377 unique studies appeared relevant. The list of titles and abstracts was reviewed, and 69 studies were selected for full-text review. Twenty-nine articles, from Canada, the UK, Australia, Austria, Denmark, Italy, Netherlands, and Norway, were analyzed for a critical narrative summary report (4;6–19;23;31–43). Included studies are described in Tables 1–4. Adherence to JBI quality checklists for all studies is presented in Table 5. The data extracted from each study are reported in additional file 2, and described in detail in the following section. Reporting quality of the single cost–utility analysis (23) was additionally assessed using the CHEERS 2022 (30) checklist and results presented in Additional File 3.

We report the findings of the quality assessment in Table 5. Of the cohort studies, adherence ranged from 73 to 91 percent of checklist items. Only one study reported strategies to deal with confounding factors, and none of the studies reported strategies to address incomplete follow-up. The only economic evaluation reported 91 percent of JBI Checklist for Economic Evaluations items, lacking generalizability of the findings to other health systems. Of the qualitative research studies, adherence ranged from 80 to 100 percent, with studies not reporting of cultural location of the researchers, or the influence of the researcher on the research. Of the three cross-sectional studies, one study met each of the quality criteria, whereas the other two studies did not identify confounding factors, or state strategies to deal with them. Only one quasi-experimental study was included, which did not clearly state the control group. These quality appraisal findings are also reported alongside the extracted information provided in Tables 1–4.

### Summary of findings on private elective surgical provision by outcome

Here we present the results of the review by the outcomes of interest – accessibility, acceptability, safety, efficiency, clinical effectiveness, and cost-effectiveness. We note that several studies did not provide detailed patient information such as ethnic group and case severity, even though this information is important to evaluate accessibility, patient selection issues, effectiveness of the treatment, and complications. This limits the generalizability of some studies and limits the conclusions that can be drawn when considering the relevance of the evidence to local decision making.

#### Accessibility

Access to health care is defined as the extent to which financial, organizational, geographical, and cultural barriers are minimized for patients (44). Eleven papers from the UK, Australia, Denmark, Canada, Netherlands, and Italy discussed the accessibility of services for cataract, hip, and knee patients and compared the private and public provision of these surgical procedures (7;12;15–18;31;33;34;39;41). Among these studies, only two (33;34) looked at accessibility related to patient preoperative/ general health status or symptom severity, and neither reached a definite conclusion about the relevance of these factors. Included studies focused on accessibility are summarized by surgery type in Table 1.

#### Acceptability

Although the included studies offer little information on defining or assessing acceptability, a theoretical framework defines the concept as a patient’s cognitive and emotional responses to the intervention (45). From a healthcare perspective, the primary acceptability indicator is satisfaction level. In seven papers, two from the UK (10;37), three from Australia (6;32;33), one from Denmark (7), and one from the Netherlands (17), the acceptability of surgical services was discussed. Studies that included an assessment of acceptability are summarized in Table 1.

#### Safety

Factors impacting patient safety in the outpatient surgery population include surgical preparedness, patient education, and clinically appropriate and accurate surgical procedures (46). Several papers addressed safety considerations for elective surgical procedures: four from the UK (8;10;11;15), two from Australia (32;38), three from Denmark (7;34;35), one from Norway (14), and one from Italy (41). Results are summarized by surgery type in Table 1.

#### Clinical effectiveness

Clinical effectiveness can be assessed by many outcomes, such as improvements life-years gained, symptom relief, patient-reported outcomes, or cure. Researchers explored postoperative outcomes, readmission rates, reoperation rates, or short-term complications of surgical procedures as a clinical effectiveness factor in eight studies (6; 8;10;11;13;18;33;42). The most extensive research on the correlation between clinical effectiveness and care provider types was conducted in the UK (8;10;11) and Australia (6;13;33;42). Results of included studies are summarized by surgery type in Table 2.

**Table 2.** Studies evaluating clinical effectiveness and efficiency

Clinical effectiveness						
Surgery type	Study	Proportion of checklist items met	Country	Population	Outcome measure	Results
Cataract	Pager and McCluskey 2004	0.80	Australia	Cataract surgery patients. Forty-two public patients and 39 private patients in Sydney, Australia between April and June 2002. Average age: 71.7 ± 9.6 (private) vs. 72.9 ± 9.4 (public)	postoperative outcomes	Comparing preoperative and postoperative VF-14 (Visual Function Index) scores, both groups achieved the same level of postoperative outcomes. The VF-14 scores reached 91.5 ± 13 and 92.8 ± 14), for private and public patients, respectively.
Orthopedic	Adie et al. 2012	0.82	Australia	Scheduled hip or knee surgery patients in Sydney, Australia from April 2007 to December 2008. (331 total, 184 public, 147 private; 215 knees, 116 hips). Average age: 68 ± 12.3 (private) vs. 68 ± 9.8 (public)	health-related quality of life scores	Magnitude and rate of improvement in Oxford score or quality of life was similar for patients treated in public and private hospitals.
	Bannister et al. 2010	0.73	UK	Hip and knee arthroplasty patients in the UK from October 2003 to March 2005. Patients who had 880 total hip and 874 total knee arthroplasties at a regional orthopedic hospital with 368 total hip and 365 total knee arthroplasties from an NHS and 67 total hip and 86 total knee arthroplasties from a private hospital independent treatment center.	readmission rates, reoperation rates	Early re-operation rates were 9% at private hospitals, 1.4% at public ones after THR and 8% at private hospitals, 1.9% at public ones after TKR. Readmission rates were 13% at private, 0.6% at public. After knee surgery, re-admission rates from private hospitals were 13%, 1% at public ones.
	Chard et al. 2011	0.82	UK	Patients undergoing hip or knee replacement (5671 in ISTCs and 14 292 in NHS), inguinal hernia repair (640 and 2023, respectively), or surgery for varicose veins (248 and 1336, respectively) in the UK from June 2008 to September 2009.	health-related quality of life scores	After adjustment for preoperative differences, patients undergoing joint replacements in NHS providers had poorer outcomes: difference of −1.7 (95% CI *–*2.5 to −0.9) on the Oxford hip score and − 0.9 (−1.6 to −0.2) on the Oxford knee score.
	Chhabra et al. 2022	0.90	Australia	Public and private patients attending a 6-week follow-up appointment after TKA at one of four clinical services in the Australian Capital Territory between 1 February 2018 and 31 January 2019. Age: Private patients aged 65–84 years (63%) compared with public (53%), whereas proportionally more public patients were in the 45–64 age group (44% compared with 35%).	readmission rates	Public patients were significantly more likely to be readmitted within 30 days compared with private patients (adjusted OR = 6.31, 95% CI: 1.59 to 25.14, p = 0.009), and patients who attended rehabilitation were significantly less likely to be readmitted within 30 days of discharge than those who did not (adjusted OR = 0.16, 95% CI: 0.04 to 0.57, p = 0.005).
	Harris et al. 2019	0.73	Australia	Patients who received primary hip and knee replacement between 2003 and 2016 in Australia. (Hip: 210 828 private, 100 931 public, Knee: 338 259 private, 160 642 public procedures.)	revision rates	Higher revision rates for total hip revision (17.4% private vs. 4.4% public) and total knee revision (19.6% private vs. 10.0% public) in private hospitals.
Both	Browne et al. 2008	0.73	UK	769 patients (inguinal hernia, varicose vein, and cataract) treated in six private and 1895 treated in 20 public facilities in England during 2006–07. Age: 73.7 ± 10.6 (cataract public), 74.6 ± 10.0 (cataract private), 66.2 ± 14.6 (hip public), 66.8 ± 14.1 (hip private), 66.2 ± 16.5 (knee public), 66.7 ± 12.2(knee private).	functional status and quality of life improvement	After adjustments for preoperative characteristics ISTCs: cataract surgery patients achieved a significantly better outcome on the VF14 (2.6 points on a 100-point scale, p = 0.005) and the EQ-5D (0.03 points on a 0 to 1 scale, p = 0.01); hip replacement patients on the Oxford Hip Scores (2.4 points on a 70-point scale, p = 0.03) and the EQ-5D (0.06 points, p = 0.03).
	Tulp et al. 2020	0.75	Netherlands	Patients who had anterior cruciate ligament surgery, cataract surgery, total hip replacement, total knee replacement, and carpal tunnel syndrome surgery in Dutch hospitals and ISTCs in 2017.	revision rates	Revision surgery after THR was performed more frequently in private facilities than in public hospitals. A regression analysis estimated a 1.44% higher revision rate in private facilities.

Efficiency						
Surgery type	Study		Country		Outcome measure	Results
Cataract	Barbieri et al. 2007	0.88	Austria	Patients admitted to hospital who were diagnosed with cataracts or underwent cataract surgeries in Austria, from 2001–2002. Public hospitals in 2001 (48 443 patients) and of public (47 797 patients) and private hospitals (5100 patients) in 2002.Age: 73.77 ± 10.86 (public hospital), 75.01 ± 9.4 (private hospital)	length of stay	The average length of stay (LOS): 4.21(4.18; 4.23) in public hospitals and 2.97 (2.91; 3.02) in private hospitals (p < .001).
	Li et al. 2004	0.75	Australia	All patients who underwent cataract surgery (117 083 procedures), along with those in whom postoperative endophthalmitis subsequently developed from 1980 to 2000 in Western Australia. Age: <80 years (63.3%), > 80 years (36.7%)	number of the surgical procedures completed in a year	More cataract surgeries were performed in private hospitals than in public hospitals and hospitals in rural areas.
Orthopedic	Cooper et al. 2018	1	UK	All elective hip and knee replacements (478 226) on patients aged 55–100 performed between financial years 2002/3 and 2008/9 in England.	length of stay	The entry of for-profit specialty surgical centres led to a 16% reduction in pre-surgery LOS at nearby public hospitals, increasing the proportion of patients treated on the day of admission.
	Kelly and Stoye 2016	0.82	UK	Elective hip replacement patients between April 2002 and March 2011 analyzed for 6 781 aggregated geographic areas.	length of stay	There is no effect of independent sector provider exposure on preoperative LOS.
	Kelly and Stoye 2020	0.82	UK	Elective hip replacement patients (615,281 patients) between April 2002 and March 2013.	length of stay, number of the surgical procedures completed in a year	A five-day decrease in median LOS (from 9 to 4 days) at public hospitals over study period. In 2012–13, median LOS was 4 days for public and private providers. Private hospital entry into publicly funded elective market increased total number of publicly funded hip replacements, did not reduce total cases in public facilities.
	Koehoorn et al. 2011	0.82	Canada	Workers with accepted workers compensation claim for meniscal injury. 1380 surgeries from 2001 to 2005 in British Columbia, Canada. Average age: 46.1 (private) vs. 44.7 (public)	time to return to work	Return to work time (median days, IQR) 60(35,162) public hospital vs. 66(37,161) private hospital.
	Siciliani et al. 2013	0.73	UK	Patients who received primary hip replacement funded by the English NHS for financial year 2006–2007. (42 948 patients, of which 1 841 were treated at 173 public treatment centers and 6 specialized treatment centers, and 938 by 14 private treatment centers). Age: 68.67 ± 10.91 (public treatment center), 69.99 ± 8.69 (private treatment center)	length of stay	Specialized public treatment centers (5.866 ± 2.572) and private treatment centers (4.481 ± 1.494) have 18% and 40% shorter LOS than public hospitals (7.455 ± 4.780), respectively.
	Vanhegan et al. 2015	0.73	UK	Orthopedic surgery patients whose provider held contracts at both a public facility in 2011 and a private facility in 2012. 66 surgeries in 2011, 32 in 2012.	number of the surgical procedures completed in a year, reduction in potential financial productivity	Same surgeon undertook 18 lists in pre- and post-ISTC years with 66 patients (pre-ISTC) and 32 (post-ISTC), eliciting a reduction in productivity of 51.5%.
Both	Street et al. 2010	0.73	UK	Patients receiving care for one of several defined healthcare resource groups (including hip and knee replacements) in the UK in financial year 2006/07. A total of 3 334 535 patients are included in the analysis, of which 77 358 (2.3%) were treated in treatment centers. Age: 69.72 ± 9.58 (knee patients), 70.66 ± 10.35 (hip patients)	length of stay	Very little difference in LOS (weighted mean difference − 0.25 days, 99% CI –0.28 to −0.22) between patients treated in hospitals and treatment centers except for hip and knee replacements. Those receiving hip and knee replacement surgeries in public facilities stayed longer than those treated in private treatment centers.
	Tulp et al. 2020	0.75	Netherlands	Patients who had anterior cruciate ligament surgery, cataract surgery, total hip replacement, total knee replacement, and carpal tunnel syndrome surgery in Dutch hospitals and ISTCs in 2017.	procedure cost	Cataract, THR and TKR procedures were performed more often in a public hospital setting, leading to lower list prices for procedures, although the effect is limited. Referenced against a general hospital the results are Cataract (−49.15(41.61); TKR 203.98(519.83); THR 460.97(465.21).

#### Efficiency

Efficiency refers to how to use resources effectively to achieve an objective (47). Surgical service efficiency was examined in ten studies (4;9;15;18;19;31;36;38–40). Most of the research adopted the technical efficiency perspective, so the authors evaluated length of stay (LOS), defined as from admission to discharge or preoperative only (4;9;15;36;39), and a number of the surgical procedures completed in a year (15;19;38). Studies on efficiency are reported in Table 2.

#### Cost and cost-effectiveness

Goodacre and McCabe define a cost-effective intervention as an intervention that represents good value for money (48). One cost–utility analysis (23) and one costing study (17) included in this review and presented in Table 3. The evidence on cost-effectiveness of introducing private providers into the publicly funded market is highly limited.

**Table 3.** Studies evaluating cost and cost-effectiveness.

Surgery type	Study	Proportion of checklist items met	Country	Population	Outcome measure	Results
Cataract	Kruse et al. 2019	0.82	Netherlands	50 000 patients who received cataract care (including academic and tertiary hospitals) from 2013 to 2015. Average age: 72.26 ± 9.77 (private) vs. 73 ± 10.1 (public)	treatment cost	Private facilities costs were 7% lower compared to public facilities (p < .01).
Orthopedic	Karnon et al. 2018	0.91	Australia	Non-urgent patients for total knee replacement surgery to a maximum age of 100 years in Australia. Used data inputs from published sources in a modelling study.	incremental cost-effectiveness ratio	With the purchase of private services, additional quality-adjusted life years (QALYs) could be gained at an incremental cost of less than 40,000 2016 Australian Dollars.

#### Patient characteristics and selection issues

Our review also identified additional important findings around the selection of patients receiving care in different facility types. Terms such as cherry-picking, cream-skimming, and dumping were used in several studies to describe approaches to patient selection by private providers (4;8–11;14;33–35;40;43).

Generally, private facilities are alleged to cherry-pick or cream-skim by selecting less complex patients, which (i) increases postoperative LOS and costs for public facilities, (ii) restricts access to private facilities for certain groups of patients, and (iii) increases inequality within the health system (10;11;34). Dumping occurs when patients from private facilities are referred to public facilities in the event of adverse surgical outcomes (35).

Fourteen papers compared the characteristics of patients. They found that patients who have surgery in private hospitals are healthier (10;11;34) and younger (14;34;40) than those who have surgery in public hospitals. Private hospital patients also have fewer comorbidities (10;14) and less severe preoperative symptoms (4;10;11;33;43). The included studies are summarized by surgery type in Table 4.

**Table 4.** Studies evaluating patient selection issues

Surgery type	Study	JBI quality score	Country	Population	Outcome measure	Results
Cataract	Barbieri et al. 2007	0.88	Austria	Patients admitted to hospital who were diagnosed with cataracts or underwent cataract surgeries in Austria, from 2001–2002. Public hospitals in 2001 (48 443 patients) and of public (47 797 patients) and private hospitals (5100 patients) in 2002. Age: 73.77 ± 10.86 (public hospital), 75.01 ± 9.4 (private hospital)	complex patients	Public hospitals often did additional procedures during one staying period (8773 vs. 1337). The rates for cataract intervention in both eyes and for one single intervention were 7.01% (0.75; 13.29) and 92.98% (86.71; 99.25) in public hospitals. In private hospitals, these rates were 2.47% (0; 5.91) and 97.53% (94.09; 100).
	Browne et al. 2008	0.73	UK	769 patients (inguinal hernia, varicose vein, and cataract) treated in six private and 1895 treated in 20 public facilities in England during 2006–07. Age: 73.7 ± 10.6 (cataract public), 74.6 ± 10.0 (cataract private), 66.2 ± 14.6 (hip public), 66.8 ± 14.1 (hip private), 66.2 ± 16.5 (knee public), 66.7 ± 12.2(knee private).	complex patients	Patients undergoing day surgery in ISTCs were healthier and had a less severe primary condition than those in NHS facilities. Poor or fair health mean (SD): cataract, NHS: 131 (22.3), ISTC 45(16.4). Hip replacement, NHS: 62(21.5), ISTC 27(14.9). Knee replacement, NHS 68(21.7), ISTC 21(11.5).
	Kruse et al. 2019	0.82	Netherlands	50 000 patients who received cataract care (including academic and tertiary hospitals) from 2013 to 2015. Average age: 72.26 ± 9.77 (private) vs. 73 ± 10.1 (public)	complex patients	The mean age is lower in private clinics, 72.26(9.77) vs. 73.20(10.10). The percentage of patients who are 85 years or older is much lower in private clinics (8.32%) than in general hospitals (10.29%). The average number of chronic conditions shows that private patients have less comorbidity, 2.15(1.65) vs. 2.24(1.72).
	Pager and McCluskey 2004	0.80	Australia	Cataract surgery patients. Forty-two public patients and 39 private patients in Sydney, Australia between April and June 2002. Average age: 71.7 ± 9.6 (private) vs. 72.9 ± 9.4 (public)	complex patients	Preoperative VF-14 scores were 86.5 ± 11.7 for private patients and 79.0 ± 19 for public patients (p = 0.035).
	Solborg et al. 2015	0.73	Denmark	Cataract Surgery patients in Denmark between 2004 and 2012. 243 856 patients (411 140 cataract operations).	complex patients	The mean age at first eye cataract surgery in private hospitals/clinics was 0.61 years lower compared to the mean age at first eye cataract surgery in public hospitals (95% CI: 0.36–0.87, p < .001). Patients who had cataract surgery in private hospitals were healthier.
	Solborg et al. 2013	0.73	Denmark	Patients who received primary hip replacement funded by the English NHS for financial year 2006–2007. (42 948 patients, of which 1 841 were treated at 173 public treatment centers and 6 specialized treatment centers, and 938 by 14 private treatment centers).	postoperative infection treatment	All endophthalmitis cases after cataract surgery (36% performed at public hospitals and 64% performed at private hospitals) were treated in public hospitals.
Orthopedic	Bannister et al. 2010	0.73	UK	Hip and knee arthroplasty patients in the UK from October 2003 to March 2005. Patients who had 880 total hip and 874 total knee arthroplasties at a regional orthopedic hospital with 368 total hip and 365 total knee arthroplasties from an NHS and 67 total hip and 86 total knee arthroplasties from a private hospital independent treatment center.	patient rejection (complexity of the surgery or comorbidity)	Reasons for rejection at public hospitals were co-morbidity in 4.2% and complexity of surgery in 1%. At private hospitals, 23.2% were rejected on medical or surgical grounds.
	Chard et al. 2011	0.82	UK	Patients undergoing hip or knee replacement (5671 in ISTCs and 14 292 in NHS), inguinal hernia repair (640 and 2023, respectively), or surgery for varicose veins (248 and 1336, respectively) in the UK from June 2008 to September 2009.	complex patients	Patients in ISTCs were healthier than those in NHS providers, had less severe preoperative symptoms, and were more affluent, though the differences were small.
	Cooper et al. 2018	1	UK	All elective hip and knee replacements (478 226) on patients aged 55–100 performed between financial years 2002/3 and 2008/9 in England.	complex patients	ISTC entry led nearby public hospitals to experience an 11.6% increase in patients’ average illness severity as captured by the Charlson score – or a 6.2 percentage point increase in the proportion of patients with a Charlson score of three or more.
	Heath et al. (2022)	0.82	Australia	Patients undergoing primary THR (specifically total conventional hip replacement) or TKR for osteoarthritis between July 2018 and April 2020 who participated in the AOANJRR PROMs program (4330 THR patients and 7054 TKR patients). Mean age: 66.48 ± 11.28 (public hip replacement patients), 67.01 ± 11.28 (private hip replacement patients), 66.17 ± 9.08 (public knee replacement patients), 66.57 ± 8.43 (private knee replacement patients)	Preoperative symptom severity	The preoperative estimated mean Oxford Hip Score was significantly higher (reflecting less symptoms) for patients having surgery in private versus public hospitals (21.39 versus 18.11, [mean difference 3.27, 95% CI 1.81, 4.79]). For TKR, there was a significant interaction between BMI and hospital type and a significant interaction between gender and hospital type where the largest difference in Oxford Knee Score between private and public hospitals was seen among underweight/normal BMI patients (mean difference of 4.68, 95% CI 2.99, 6.37).
	Holom and Hagen 2017	0.91	Norway	All publicly financed patients having primary total hip (37 897 patients) or primary total knee arthroplasty (25 802 patients) at one of the three hospital types from 2009 to 2014 in Norway. Average age: 66.07 ± 10.18 (private) vs. 68.26 ± 11.20 (public)	complex patients	Patients who had surgery at private facilities had the lowest average Charlson Comorbidity Index. Mean, SD private (0.116, 0.4340 vs. public (0.263, 0.798).
	Kelly and Stoye 2020	0.82	UK	Elective hip replacement patients (615,281 patients) between April 2002 and March 2013.	comorbidity	The mean number of comorbidities was 1.83(0.6) for patients treated in private facilities and 3.10(1.86) for the ones treated in NHS hospitals.
	Moscone et al. 2019	0.73	Italy	Acute myocardial infarction, stroke, hip and knee replacement patients admitted to 189 Italian hospitals located in the Lombardy region between 2012 and 2014. Age: 68.25 (hip replacement), 70.12 (knee replacement)	complex patients	Severe patients with a higher Elixhauser comorbidities index who require hip and knee surgery were less likely to be admitted to private facilities. Hip replacement, Elixhauser comorbidities index 0.023(0.3866) private vs. public 0.116(0.386). Knee replacement, Elixhauser comorbidities index 0.030(0.204) private vs. 0.135(0.421) public.
Both	Street et al. 2010	0.73	UK	Patients receiving care for one of several defined healthcare resource groups (including hip and knee replacements) in the UK in financial year 2006/07. A total of 3 334 535 patients are included in the analysis, of which 77 358 (2.3%) were treated in treatment centers. Age: 69.72 ± 9.58 (knee patients), 70.66 ± 10.35 (hip patients)	complex patients	Patients treated in public centers were younger, more likely to have come from deprived areas, and tended to have more diagnostic and procedure codes than those treated in private centers.

**Table 5.** The quality measures of included studies based on the Joanna Briggs Institute quality appraisal checklist

The quality measures of Cohort studies based on the JBI quality appraisal checklist													
Author	Criteria and corresponding scores												
	#1	#2	#3	#4	#5	#6	#7	#8	#9	#10	#11	Total	%
Adie et al.	1	1	1	1	1	1	1	1	0	0	1	9/11	0.82
Bannister et al.	1	1	1	0	0	1	1	1	1	0	1	8/11	0.73
Browne et al.	1	1	1	0	0	1	1	1	1	0	1	8/11	0.73
Chard et al.	1	1	1	1	0	1	1	1	1	0	1	9/11	0.82
Harris et al.	1	1	1	0	0	1	1	1	1	0	1	8/11	0.73
Heath et al.	1	1	1	0	1	1	1	1	1	0	1	9/11	0.82
Holom et al.	1	1	1	1	1	1	1	1	1	0	1	10/11	0.91
Kelly 2016	1	1	1	1	0	1	1	1	1	0	1	9/11	0.82
Kelly 2020	1	1	1	1	0	1	1	1	1	0	1	9/11	0.82
Kirkwood	NA	NA	1	1	1	1	1	1	1	NA	1	8/8	1.0
Koehoorn et al.	1	1	1	1	0	1	1	1	1	0	1	9/11	0.82
Kruse et al.	1	1	1	1	0	1	1	1	1	0	1	9/11	0.82
Moscone et al.	1	1	1	0	0	1	1	1	1	0	1	8/11	0.73
Siciliani et al.	1	1	1	0	0	1	1	1	1	0	1	8/11	0.73
Solborg Bjerrum et al. 2013	1	1	1	0	0	1	1	1	1	0	1	8/11	0.73
Solborg Bjerrum et al. 2015	1	1	1	0	0	1	1	1	1	0	1	8/11	0.73
Street et al.	1	1	1	0	0	1	1	1	1	0	1	8/11	0.73
Vanhegan et al.	1	1	1	0	0	1	1	1	1	0	1	8/11	0.73

The quality measures of economic evaluations based on the JBI quality appraisal checklist													
Author	Criteria and corresponding scores												
	#1	#2	#3	#4	#5	#6	#7	#8	#9	#10	#11	Total	%
Karnon et al.	1	1	1	1	1	1	1	1	1	1	0	10/11	0.91

The quality measures of qualitative research studies based on the JBI quality appraisal checklist												
Author	Criteria and corresponding scores											
	#1	#2	#3	#4	#5	#6	#7	#8	#9	#10	Total	%
Andersen and Jacobsen	1	1	1	1	1	0	0	1	1	1	8/10	0.80
Chhabra et al.	1	1	1	1	1	1	0	1	1	1	9/10	0.90
Fitzpatrick et al.	1	1	1	1	1	0	0	1	1	1	8/10	0.80
Naylor et al.	1	1	1	1	1	1	1	1	1	1	10/10	1
Pager et al.	1	1	1	1	1	0	0	1	1	1	8/10	0.80
Perotin et al.	1	1	1	1	1	0	0	1	1	1	8/10	0.80

The quality measures of analytical cross-sectional studies based on the JBI quality appraisal checklist										
Author	Criteria and corresponding scores									
	#1	#2	#3	#4	#5	#6	#7	#8	Total	%
Cooper et al.	1	1	1	1	1	1	1	1	8/8	1
Li et al.	1	1	1	1	0	0	1	1	6/8	0.75
Tulp et al.	1	1	1	1	0	0	1	1	6/8	0.75

The quality measures of quasi-experimental studies based on the JBI quality appraisal checklist											
Author	Criteria and corresponding scores										
	#1	#2	#3	#4	#5	#6	#7	#8	#9	Total	%
Barbieri et al.	1	1	1	0	1	1	1	1	1	8/9	0.88

“1” indicates the study does fulfill the specified criteria, whereas “0” indicates the study does not fulfill the stated criteria. NA indicates that the criteria were not applicable to the study.

### Summary of findings on private elective surgical provision by country

#### Austria

One study from Austria (9) compared the LOS and found that private hospitals had a significantly shorter average LOS than public hospitals. The authors explained the difference by stating that public hospitals often performed additional procedures during one admission. Study results showed that the rates for cataract intervention in both eyes were nearly three times higher in public hospitals.

#### Australia

There are eight Australian studies that looked at various outcomes to compare the public and private surgical facilities for cataract and orthopedic surgeries. One study (38) compared surgical volumes found that more cataract surgeries were performed in private hospitals than in public hospitals.

The only Australian study (33) assessed surgical wait times and found that public hospitals were nine times longer than private center patients. Studies that evaluate patient satisfaction levels have conflicting results. In some studies, public-sector patients were less satisfied overall than private-sector patients (33), in others (6) private patients were less likely to be satisfied due to their higher expectations, whereas some did not find a statistically significant difference Naylor et al (32).

Similar to patient satisfaction, postoperative outcome results were mixed. In some studies, procedures performed in private hospitals had a significantly higher risk of postoperative complication (38) and revision rates (13) than those performed in public hospitals, whereas others show that both groups achieved the same level of postoperative outcomes (6;33). One study (32) states the complication rates were different for hip arthroplasty but were the same for knee arthroplasty. It is worth noting that the variation in revision rates after hip and knee surgeries could be mainly due to differences in prosthesis selection (13). One study found that following total knee arthroplasty, public patients were significantly more likely to be readmitted within 30 days compared with private patients and the authors concluded that these higher readmission rates might be explained by several contributing factors such as socioeconomic status, longer waiting times resulting in increased impairment and disease complexity (42). A recently published study evaluated the preoperative symptom severity and found that patients who underwent primary total hip replacement or total knee replacement for osteoarthritis had significantly worse preoperative symptom severity if their surgery was performed at a public hospital which may reflect variation in access to surgery, and surgeon and patient preferences between these groups (43).

The only study accessed the cost-effectiveness of contracting with the private sector for TKR and found that with the purchase of private services, additional quality-adjusted life years (QALYs) could be gained at an incremental cost of less than 40,000 2016 Australian Dollars (23).

#### Canada

Some workers’ compensation systems in Canada pay higher fees to expedited surgeries to reduce surgery wait times, disability costs and improve return-to-work outcomes. Policies vary among provinces, but in this approach, clinics are expected to perform expedited operations within 21 days of surgery decision. A study (31) assessed the effect of expedited surgical fees and revealed that the public expedited group had the shortest disability duration from surgical consult to return to work around one workweek.

#### Denmark

Studies from Denmark show that private clinics reduce wait times more than public clinics (7), and private facilities offer second eye surgery sooner than public facilities (34). One study (7) reported higher patient satisfaction with private clinics after hip replacements. On the other hand, due to high complication (7;35), and overall mortality rates (34) in public hospitals, dumping and patient selection are concerns that are mentioned in almost all studies (7;34;35).

#### Italy

A study from Italy (41) showed that private hospitals treat more hip and knee replacement patients with a shorter wait time, but with higher 30-day emergency readmission rates. Similar to studies from Denmark, this study also highlighted that severe patients were less likely to be admitted to private facilities either due to a lack of facilities to treat the patients with a high comorbidity index, specialization in routine cases, or a combination of dumping and cherry-picking (41).

#### The Netherlands

Two studies from the Netherlands had information on the predefined outcomes. One study found that ISTCs cataract surgery volume is slightly higher than in general hospitals. They identified significantly higher patient satisfaction scores among ISTC patients compared with public hospital patients with a lower cost (17). The main reasons for the lower cost were to perform less severe patients’ cataract surgeries and claim the fewer care activities, more intense optometrist use, and lower overhead costs (17). In contrast, Tulp et al. (18) showed that cataract, hip, and knee surgeries were performed more frequently in public hospitals, but revision surgery after THR was performed more frequently in private facilities than in public hospitals. It is important to note that these quality differences were not consistent over all elective surgery types and providers. ISTCs performed worse for both TKR and THR, yet outperformed public hospitals for cataract treatment.

#### Norway

Holom and Hagen (14) evaluated publicly financed primary total hip and total knee replacement patients and found that private hospitals had significantly lower rates for 30-day readmission due to complications. The authors suggest that this may be because public hospitals receive more readmissions and play a critical role in the care of more complex cases.

#### The United Kingdom

Reforms in the UK introduced in 2006 allowed ISTCs to operate within the UK health system. From 2006 onward, private hospitals were also allowed to enter the existing elective surgical treatment system and compete with the ISTCs and public hospitals (15) for publicly funded treatments.

The introduction of private-sector providers reduced waiting times at National Health Service (NHS) hospitals without any effect on surgical volumes (39), with significantly lower median wait times at private hospitals than in public hospitals (12;15). One study warned about the negative consequences of private-sector provision on equitable access to care (16) as the results showed that inequalities by age and socioeconomic deprivation were found to increase with a private provision especially for the patients aged 85 years and over and those living in more socioeconomically deprived areas.

Patient satisfaction levels were mixed in the UK studies. Some studies reported roughly equivalent satisfaction levels (10), whereas others (37) showed that public sector hospitals provided better information and more choice, whereas private-sector facilities offered a more comfortable, friendly, and clean environment.

Studies also show mixed results for postoperative outcomes and complication rates. Some studies found that patients treated in ISTCs were less likely to report postoperative problems than those treated in NHS facilities (10;11), and improvements were greater in patients treated in private centers (10;11) which could be due to patient selection (10;11). In contrast, some studies reported lower complication and reoperation rates for surgeries performed in the NHS hospital (8). Similar to studies form other countries, UK studies also state that a possible reason for the better outcomes could be that these facilities admitted healthier patients or patients who had less severe conditions than those undergoing surgery in NHS providers (11).

Studies from the UK on efficiency also report varied results. Some studies (39) did not find any effect of independent sector provider exposure on preoperative LOS, others (4;15) found that hospitals located in more competitive markets were more successful in decreasing LOS.

The introduction of ISTCs resulted in a reduction in departmental efficiency and financial productivity (19). Postoperative LOS for those receiving hip and knee replacement surgeries in public facilities was longer than for those treated in private treatment centers (36;40).

In the 2000s, after the implementation of for-profit and not-for-profit healthcare providers in the UK, public sector providers faced a staff shortage. At the same time, private centers took on less problematic patients and left the others to the public healthcare providers (4). As a result, the public sector had to deal with more complex cases, comorbidities, and complications with fewer staff. Case selection issues were evaluated by Bannister et al. (8). In contrast to previous studies (10), they found that one ISTC rejected referred surgical cases due to either the complexity of the surgery or associated co-morbidities. Studies found that patients treated in public centers were younger, more likely to have come from deprived areas, and tended to have more diagnostic and procedure codes than those treated in private centers (11;15;40). Discussions around the potential for shifting such patients to the public system raise another concern regarding private-sector provision.

### Summary of findings on private elective surgical provision by surgery type

#### Cataract surgery

Four papers from Australia, Denmark, and Netherlands discussed the accessibility of services for cataract patients and compared the private and public provision of these surgical procedures (17;18;33;34). Among these studies, only two (33;34) looked at accessibility related to patient preoperative/ general health status or symptom severity, and neither reached a definite conclusion about the relevance of these factors. Two studies looked at the surgery volume and one found that the average number of surgeries is higher within private facilities (17), and the other study stated that cataract surgeries were performed more frequently in public hospitals (18). The average number of surgeries is higher within private facilities than in general hospitals (17). Private hospitals have shorter wait times than public hospitals (33;34). Patient satisfaction levels are higher for private clinics than public clinics (10;17;33).

Studies assessed safety found that procedures performed in private hospitals had a significantly higher risk of postoperative infection than did those performed in public hospitals (35;38), whereas overall mortality in patients who had regular cataract surgery in public hospitals was higher compared to patients who had cataract surgery in private hospitals (34). Only one study evaluated the postoperative outcomes and did not find any difference between the public and private facilities (33), whereas another study showed that after adjustments for preoperative characteristics private facilities achieved significantly better outcomes (10). Private facilities were more efficient with a lower LOS (9) and a higher number of surgical procedures completed in a year than the public facilities (38). Private facility’s costs were 7 percent lower compared to public facilities (17). Patients undergoing day surgery in private facilities were healthier, younger, and had a less severe primary condition than those in public facilities (10;17;33;34;40).

#### Orthopedic surgery

Eight papers from Canada, Denmark, Italy, Netherlands, and the UK discussed the accessibility of services for orthopedic patients and compared the private and public provision of these surgical procedures (7;12;15;16;18;31;39;41). Six studies used wait times to evaluate accessibility (7;12;15;24;31;39;41), other studies used treatment inequalities (16) and surgery volume (18). Hip and knee surgeries were performed more frequently in public hospitals (18). Increased use of private-sector provision was associated with a significant decrease in direct NHS provision with widening inequalities by age or socio-economic deprivation (16). Except for one study (31), private facilities have shorter wait times than public clinics (7;12;15;41). Patient satisfaction levels are higher for private clinics than public clinics (7;10). Public sector hospitals provided better information and more choice, whereas private-sector facilities offered a more comfortable, friendly, clean environment (37)and more frequent surgeon visitation (32). Patients undergoing joint replacements in public hospitals more often reported complications (7;11;32). We found mixed results for 30-day readmission dates and quality of life improvement after orthopedic surgeries. According to two study results, private hospitals had significantly lower rates for 30-day readmission than the public hospitals (14;15), whereas three studies reported high readmission rates for private hospitals compared to the public ones (7;8;41). The revision rates were higher in private facilities both for hip (13;18) and knee surgeries (13). Private facilities have shorter LOS than public facilities (36;40). A cost–utility study showed that with the purchase of private services, additional QALYs could be gained at an incremental cost of less than 40,000 2016 Australian Dollars (23). Patients in private facilities were healthier (11;14;15;40;41)and younger (40) than those in public ones, had less severe preoperative symptoms (11;43), and were more affluent (11;40).

## Discussion

In this review, we evaluated the three main dimensions of healthcare quality alongside efficiency and cost considerations: safety, clinical effectiveness, and patient experience (49). The results of the primary studies provide a mixed picture of the outcomes for private and public provision. Some results suggest that private-sector provision has a positive impact on public health system providers’ outcomes primarily due to competition (4), and higher degrees of competition are associated with greater improvements in quality (50). Studies also show the increased quality of care in hospitals located in more competitive areas than hospitals located in less competitive areas, without increased expenditures (50;51). Although the evidence suggests that competition increases health system quality (1), price regulation mechanisms are also important. Private surgical clinics generally refuse to disclose their financial statements, making it difficult to know the extent to which inappropriate or unnecessary surgeries occur in the private sector. Theoretical models show that when delivery side competition is combined with price regulation, wait times are reduced, and patient’s quality of care increases (52). Although the included studies used various methods, only four (14;15;39;41) applied causal inference methods rather than regression-based approaches with controls.

There is limited evidence that private-sector contracts within the publicly funded health system will address existing problems of capacity and waiting times in healthcare delivery. Even though there were statistically significant reductions in wait times for patients treated in private facilities in most studies, the evidence on the importance of wait times on patient preferences is controversial. A recent Australian study (53) identified the five most important attributes that patients consider when making decisions about cataract surgery in an urban setting are surgical wait time, cost, travel time, hospital reputation, and surgeon experience. This qualitative study has two main limitations. First, it does not reflect the preferences of individuals seeking cataract surgery in rural areas of Australia. They may prioritize distance over surgical wait time. Secondly, non-English speaking participants’ results are different than those of English-speaking participants. Non-English-speaking participants indicated that they were content to wait for surgery on the condition that they did not have to pay (53). As described above, the evidence so far remains mixed. Private-sector providers are expected to be more efficient due to their ability to treat patients more quickly, but some available evidence challenges this view (54–56). Even if private-sector providers treat more patients in a given time period, implications for the relative quality, cost-effectiveness, and efficiency of such services are unclear. Opponents of private-sector provision remain concerned that private centers would engage in patient selection strategies, cherry-picking, cream-skimming, and dumping (4).

Tynkkynen and Vrangbaek (20) conducted a scoping review on public and private provision in Europe, and included four of the same studies as this review (7;34;35;37) as well as an overview of other systematic reviews (21). Their findings are partially consistent with ours. The authors found that although public hospitals treat patients with more comorbidities and complications who are older and more socioeconomically deprived, they consistently have better economic performance than the private ones. The review concluded that several studies addressing the economic effects of the private compared with the public provision of health care fail to consider quality and other operational dimensions, a critical blind spot that may influence results.

In an overview of systematic reviews, Herrera et al. (21) reviewed 5,918 studies to identify systematic reviews on the impact of different types of ownership on economic, administrative, and health-related outcomes. The authors analyzed nine systematic reviews and found that for-profit healthcare providers seem to have worse mortality outcomes than their not-for-profit and public counterparts. They concluded that substantial evidence gaps in the literature remain in the comparison between public and private-sector providers. The number of economic evaluation studies was considerably smaller, so future research could substantially contribute to the economic impacts of introducing private surgical facilities in publicly funded healthcare systems.

### Limitations

This systematic review has several limitations. We have restricted our search to studies published after January 2000 and only included English language publications. Although limiting searches to English-only studies is common, this could cause “English-language bias” and limit the generalizability of the results. Also, detailed patient information such as ethnic group and case severity was not given in all included studies, even though this information is important to evaluate accessibility, patient selection issues, effectiveness of the treatment, and complications. Although this is a limitation of the evidence, rather than our study design, it does limit the conclusions that can be drawn. Finally, this study is limited to a relatively narrow set of procedures - future studies could be designed to evaluate other surgical interventions or systems with a broader mix of public and private healthcare delivery options to provide a broader perspective.

It is important to note that system structure and payment models have a role to play in determining how private providers participate in a given market, but this is outside of the aim of this review. Our aim is to identify evidence for differences in outcomes between public and private providers, given that a public payer commissions services from both public and privately operated care providers. Providing recommendations on how private capacity can be used in any given health system, payment models, or health system financing was out of scope, but could be valuable for future research.

## Conclusions

The evidence we identified on accessibility, acceptability, safety, efficiency, and clinical effectiveness does not show a clear advantage of one delivery model over another. Rather there are strengths and weaknesses for both models. Decision-makers should take into account the evidence presented above and assess it against current and anticipated system needs when considering the role of private providers in publicly funded settings. The use of privately operated facilities to perform publicly funded services is sometimes proposed as an alternative way to deliver health care more efficiently in resource-constrained systems, though in our review the outcomes with the most limited evidence base were costs and cost-effectiveness. As economic arguments are often used in public debates on the use of private providers, further research on these outcomes is warranted.

## Supplementary material

For supplementary material accompanying this paper visit https://doi.org/10.1017/S0266462323000120. click here to view supplementary material

## References

[1] Gaynor M, Moreno-Serra R, Propper C. Can competition improve outcomes in uk health care? Lessons from the past two decades. J Health Serv Res Policy. 2012;17(S1):49–54. 22315477 10.1258/jhsrp.2011.011019

[2] Bachelet VC, Goyenechea M, Carrasco VA. Policy strategies to reduce waiting times for elective surgery: A scoping review and evidence synthesis. Int J Health Plann Manage. 2019;34:e995. 30793372 10.1002/hpm.2751

[3] Rotenberg B. Moving surgical care out of hospitals to reduce wait times. CMAJ. 2021;193(4):E138. 33667183 10.1503/cmaj.77461 PMC7954561

[4] Cooper Z, Gibbons S, Skellern M. Does competition from private surgical centres improve public hospitals’ performance? Evidence from the english national health service. J Public Econ. 2018;166:63–80.

[5] Moscelli G, Gravelle H, Siciliani L. Market structure, patient choice and hospital quality for elective patients. York, UK: Centre for Health Economics, University of York; 2016. Available from: https://www.york.ac.uk/media/che/documents/papers/researchpapers/CHERP139_market_structure_patient_choice_hospital_quality.pdf.

[6] Adie S, Dao A, Harris IA, Naylor JM, Mittal R. Satisfaction with joint replacement in public versus private hospitals: A cohort study. ANZ J Surg. 2012;82(9):616–624. 22834486 10.1111/j.1445-2197.2012.06113.x

[7] Andersen LB, Jakobsen ML. Does ownership matter for the provision of professionalized services? Hip operations at publicly and privately owned clinics in Denmark. Public Adm. 2011;89(3):956–974. 22165152 10.1111/j.1467-9299.2010.01881.x

[8] Bannister G, Ahmed M, Bannister M, Bray R, Dillon P, Eastaugh-Waring S. Early complications of total hip and knee replacement: A comparison of outcomes in a regional orthopaedic hospital and two independent treatment centres. Ann R Coll Surg Engl. 2010;92(7):610–614. 20557685 10.1308/003588410X12699663904312 PMC3229356

[9] Barbieri V, Schmid E, Ulmer H, Pfeiffer KP. Health care supply for cataract in Austrian public and private hospitals. Eur J Ophthalmol. 2007;17(4):557–564. 17671931 10.1177/112067210701700413

[10] Browne J, Jamieson L, Lewsey J, van der Meulen J, Copley L, Black N. Case-mix & patients’ reports of outcome in independent sector treatment centres: Comparison with NHS providers. BMC Health Serv Res. 2008;8:78. 18400096 10.1186/1472-6963-8-78 PMC2329629

[11] Chard J, Kuczawski M, Black N, van der Meulen J, Committee OAS. Outcomes of elective surgery undertaken in independent sector treatment centres and nhs providers in England: Audit of patient outcomes in surgery. BMJ. 2011;343:d6404. 22012180 10.1136/bmj.d6404 PMC3198262

[12] Fitzpatrick R, Norquist JM, Reeves BC, Morris RW, Murray DW, Gregg PJ. Equity and need when waiting for total hip replacement surgery. J Eval Clin Pract 2004;10(1):3–9. 14731146 10.1111/j.1365-2753.2003.00448.x

[13] Harris I, Cuthbert A, Lorimer M, de Steiger R, Lewis P, Graves SE. Outcomes of hip and knee replacement surgery in private and public hospitals in Australia. ANZ J Surg. 2019;08:08. 10.1111/ans.15154 31069924

[14] Holom G, Hagen T. Quality differences between private for-profit, private non-profit and public hospitals in Norway: A retrospective national register-based study of acute readmission rates following total hip and knee arthroplasties. BMJ Open. 2017;7(8):e015771. 10.1136/bmjopen-2016-015771 PMC5724080 28821517

[15] Kelly E, Stoye G. The impacts of private hospital entry on the public market for elective care in England. J Health Econ. 2020;73:102353. 32702512 10.1016/j.jhealeco.2020.102353

[16] Kirkwood G, Pollock AM. Patient choice and private provision decreased public provision and increased inequalities in Scotland: A case study of elective hip arthroplasty. J Pub Health. 2017;39(3):593–600. 10.1093/pubmed/fdw060 27474759

[17] Kruse F, Groenewoud S, Atsma F, van der Galien O, Adang E, Jeurissen PP. Do independent treatment centers offer more value than general hospitals? The case of cataract care. Health Serv Res. 2019;54(6):1357–1365. 31429482 10.1111/1475-6773.13201 PMC6863231

[18] Tulp A, Kruse F, Stadhouders N, Jeurissen P. Independent treatment centres are not a guarantee for high quality and low healthcare prices in the Netherlands - A study of 5 elective surgeries. Int J Health Policy Manag. 2020;9(9):380–389. 32610739 10.15171/ijhpm.2019.144 PMC7557426

[19] Vanhegan I, Hakmi A, de Roeck N, Rumian A. Effect of an independent-sector treatment centre on provision of elective orthopaedic surgery in east and north Hertfordshire. Ann R Coll Surg Engl. 2015;97(7):519–525. 26414362 10.1308/rcsann.2015.0029 PMC5210142

[20] Tynkkynen LK, Vrangbaek K. Comparing public and private providers: A scoping review of hospital services in Europe. BMC Health Serv Res. 2018;18(1):141. 29482564 10.1186/s12913-018-2953-9 PMC5828324

[21] Herrera C, Rada G, Kuhn-Barrientos L, Barrios X. Does ownership matter? An overview of systematic reviews of the performance of private for-profit, private not-for-profit and public healthcare providers. PLoS ONE. 2014;9(12):1–18. 10.1371/journal.pone.0093456 PMC4249790 25437212

[22] Canadian Institute for Health Information (CIHI). Wait times for priority procedures in Canada. Accessed on: February 7, 2022. [Internet]c2021 [updated June 15, 2021 cited February 7, 2022]. Available from: https://www.cihi.ca/en/wait-times-for-priority-procedures-in-canada.

[23] Karnon J, Haghighi B, Sajjad B, Yem S, Gamage A, Thorpe A. Cost-utility analysis of private contracting to reduce public waiting times for joint replacement surgery. Int J Technol Assess Health Care. 2018;34(2):147–155. 29455686 10.1017/S0266462317004524

[24] Canadian Institute for Health Information (CIHI). high-volume IMD procedures, 2018-2019. Accessed on: February 7, 2022. [Internet] [updated August 27, 2020]. Available from: https://www.cihi.ca/en/implantable-medical-devices-imd-in-canada.

[25] Joanna Briggs Institute (JBI). Checklist for analytical cross sectional studies [Internet]: JBI; c2020 [cited December 1, 2022]. Available from: https://jbi.global/sites/default/files/2021-10/Checklist_for_Analytical_Cross_Sectional_Studies.docx.

[26] Joanna Briggs Institute (JBI). Checklist for qualitative research [Internet]: JBI; c2020 [cited December 1, 2022]. Available from: https://jbi.global/sites/default/files/2021-10/Checklist_for_Qualitative_Research.docx.

[27] Joanna Briggs Institute (JBI). Checklist for economic evaluations [Internet]: JBI; c2020 [cited December 1, 2022]. Available from: https://jbi.global/sites/default/files/2021-10/Checklist_for_Economic_Evaluations.docx.

[28] Joanna Briggs Institute (JBI). Checklist for cohort studies [Internet]: JBI; c2020 [cited December 1, 2022]. Available from: https://jbi.global/sites/default/files/2021-10/Checklist_for_Cohort_Studies.docx.

[29] Joanna Briggs Institute (JBI). Checklist for quasi-experimental studies [Internet]: JBI; c2020 [cited December 1, 2022]. Available from: https://jbi.global/sites/default/files/2021-10/Checklist_for_Quasi-Experimental_Appraisal_Tool%20%281%29.docx.

[30] Husereau D, Drummond M, Augustovski F, de Bekker-Grob E, Briggs AH, Carswell C, et al. Consolidated health economic evaluation reporting standards 2022 (cheers 2022) statement: Updated reporting guidance for health economic evaluations. Clin Ther. 2022;44(2):158–168. 35168801 10.1016/j.clinthera.2022.01.011

[31] Koehoorn M, McLeod C, Fan J, McGrail K, Barer M, Cote P, et al. Do private clinics or expedited fees reduce disability duration for injured workers following knee surgery? Healthc Policy. 2011;7(1):55–70. 22851986 PMC3167568

[32] Naylor JM, Descallar J, Grootemaat M, Badge H, Harris I, Simpson G, et al. Is satisfaction with the acute-care experience higher amongst consumers treated in the private sector? A survey of public and private sector arthroplasty recipients. PLoS One. 2016;11(8):e0159799. 27490358 10.1371/journal.pone.0159799 PMC4973896

[33] Pager C, McCluskey P. Public versus private patient priorities and satisfaction in cataract surgery. Clin Exp Ophthalmol. 2004;32(5):482–487. 15498059 10.1111/j.1442-9071.2004.00868.x

[34] Solborg Bjerrum S, Mikkelsen K, la Cour M. Epidemiology of 411 140 cataract operations performed in public hospitals and private hospitals/clinics in Denmark between 2004 and 2012. Acta Opthalmol. 2015;93(1):16–23. 10.1111/aos.12576 25495244

[35] Solborg Bjerrum S, Kiilgaard J, Mikkelsen K, la Cour M. Outsourced cataract surgery and postoperative endophthalmitis. Acta Opthalmol. 2013;91(8):701–708. 10.1111/aos.12279 24251421

[36] Siciliani L, Sivey P, Street A. Differences in length of stay for hip replacement between public hospitals, specialised treatment centres and private providers: Selection or efficiency? Health Econ. 2013;22(2):234–242. 22223593 10.1002/hec.1826

[37] Perotin V, Zamora B, Reeves R, Bartlett W, Allen P. Does hospital ownership affect patient experience? An investigation into public-private sector differences in England. J Health Econ. 2013;32(3):633–646. 23579025 10.1016/j.jhealeco.2013.03.003

[38] Li J, Morlet N, Ng J, Semmens J, Knuiman M. Significant nonsurgical risk factors for endophthalmitis after cataract survey: EPSWA fourth report. Invest Ophthalmol Vis Sci. 2004;45(5):1321–1328. 15111584 10.1167/iovs.03-1000

[39] Kelly E, Stoye G. *New joints: Private providers and rising demand in the English National Health Service.* Institute for Fiscal Studies, IFS Working Papers: W16/15; 2016 [cited March 20, 2019]. Available from: https://www.ifs.org.uk/uploads/publications/wps/WP201615.pdf.

[40] Street A, Sivey P, Mason A, Miraldo M, Siciliani L. Are English treatment centres treating less complex patients? Health Policy. 2010;94(2):150–157. 19836851 10.1016/j.healthpol.2009.09.013

[41] Moscone F, Siciliani L, Tosetti E, Vittadini G. Do public and private hospitals differ in quality? Evidence from Italy. Reg Sci Urban Econ. 2020;83:103523.

[42] Chhabra M, Perriman D, Phillips C, Parkinson A, Glasgow N, Douglas K, et al. Understanding factors affecting 30-day unplanned readmissions for patients undergoing total knee arthroplasty (TKA): The ACT transition from hospital to home orthopaedics survey. BMJ Open. 2022;12(4):e053831. 10.1136/bmjopen-2021-053831 PMC9003601 35410923

[43] Heath EL, Ackerman IN, Holder C, Lorimer MF, Graves SE, Harris IA. Between-hospital and between-surgeon variation in thresholds for hip and knee replacement. ANZ J Surg. 2022;92(9):2229–2234. 35642256 10.1111/ans.17811

[44] Gulliford M, Figueroa-Munoz J, Morgan M, Hughes D, Gibson B, Beech R, et al. What does’ access to health care’ mean? J Health Serv Res Policy. 2002;7(3):186–188. 12171751 10.1258/135581902760082517

[45] Sekhon M, Cartwright M, Francis J. Acceptability of healthcare interventions: An overview of reviews and development of a theoretical framework. BMC Health Serv Res. 2017;17(1):88. 28126032 10.1186/s12913-017-2031-8 PMC5267473

[46] Hundt A, Carayon P, Springman S, Smith M, Florek K, Sheth R, et al. Outpatient surgery and patient safety- the patient’s voice. In: Henriksen K, Battles JB, Marks ES, Lewin DI, editors. Advances in patient safety: From research to implementation (volume 4: Programs, tools, and products). Rockville, MD: Advances in Patient Safety; 2005.

[47] CIHI. *Health system efficiency in Canada: Why does efficiency vary among regions?* April 2014 [cited June 25, 2019]. Available from: https://www.cihi.ca/en/hse_short_aib_10apr14_en.pdf.

[48] Goodacre S, McCabe C. An introduction to economic evaluation. Emerg Med J. 2002;19(3):198–201. 11971826 10.1136/emj.19.3.198 PMC1725884

[49] Doyle C, Lennox L, Bell D. A systematic review of evidence on the links between patient experience and clinical safety and effectiveness. BMJ Open. 2013;3(1):e001570. 10.1136/bmjopen-2012-001570 PMC3549241 23293244

[50] Cooper Z, Gibbons S, Jones S, McGuire A. Does hospital competition save lives? Evidence from the English NHS patient choice reforms. Econ J (London). 2011;121(554):F228–F260. 10.1111/j.1468-0297.2011.02449.x PMC4373154 25821239

[51] Gaynor M, Moreno-Serra R, Propper C. Death by market power: Reform, competition and patient outcomes in the National Health Service. *CEPR Discussion Papers;* 2011.

[52] Propper C. Competition in health care: Lessons from the English experience. Health Econ Policy Law. 2018;13(3-4):492–508. 29417915 10.1017/S1744133117000494

[53] Gilbert C, Keay L, Palagyi A, Do V, McCluskey P, White A, et al. Investigation of attributes which guide choice in cataract surgery services in Urban Sydney, Australia. Clin Exp Optom. 2018;101(3):363–371. 29345003 10.1111/cxo.12653

[54] Kruse F, Stadhouders N, Adang E, Groenewoud S, Jeurissen P. Do private hospitals outperform public hospitals regarding efficiency, accessibility, and quality of care in the European Union? A literature review. Int J Health Plann Manage 2018;33(2):434. 29498430 10.1002/hpm.2502 PMC6033142

[55] Modi N, Clarke J, McKee M. Health systems should be publicly funded and publicly provided. BMJ (Clinical Research Ed). 2018;362:k3580. 10.1136/bmj.k3580 30201731

[56] Vengberg S, Fredriksson M, Winblad U. Patient choice and provider competition - quality enhancing drivers in primary care? Soc Sci Med. 2019;226:217–224. 30878640 10.1016/j.socscimed.2019.01.042

