# Supplementary material for: A systematic review of the accessibility, acceptability, safety, efficiency, clinical effectiveness, and cost-effectiveness of private cataract and orthopedic surgery clinics
Source: Int J Technol Assess Health Care. 2023 Aug 1;39(1):e47. doi: 10.1017/S0266462323000120 (PMC11570012; doi:10.1017/S0266462323000120)
Supplement: Supplementary file 1 [file S0266462323000120sup.zip › S0266462323000120sup001.docx]

**Private Cataract/Osteopathic Surgery Services Search Strategy**

Original Search: Dagmara Chojecki, MLIS

First Search update: Lisa Tjovold, MLIS

Second Search Update: Dagmara Chojecki, MLIS

| **Database** | **Edition or Date Searched** | **Search Terms** |
| --- | --- | --- |
| **Ovid MEDLINE(R) and Epub Ahead of Print, In-Process & Other Non-Indexed Citations and Daily** 1946 to May 14, 2019 | Searched: 2019-05-14  Results: 423 | **# Searches**  1 Hospitals, Private/ or Public-Private Sector Partnerships/ or exp Private Sector/ or exp Health Facilities, Proprietary/ or exp Contract Services/  2 (proprietary or privat* or "for profit" or non-hospital or oursourc* or contract or contracts or contracting or ambulatory).ti,kf.  3 1 or 2  4 (surgery or surgeries or surgical).mp.  5 Cataract Extraction/  6 cataract*.mp.  7 (orthopedic* or hip or hips or knee*).mp.  8 Orthopedics/  9 or/5-8  10 3 and 4 and 9  11 limit 10 to (english language and yr="2000 -Current") |
| 1946 to March 25, 2021 | Search Update:  2021-03-26  Results: 158 |  |
| 1946 to October 03, 2022 | Search Update:  2021-10-03  Results: 165 |  |
| **Ovid Embase** 1974 to 2019 May 14 | Searched: 2019-05-14  Results: 584 | # Searches  1 private hospital/ or private sector/ or public-private partnership/  2 (proprietary or privat* or "for profit" or non-hospital or oursourc* or contract or contracts or contracting or ambulatory).ti,kw.  3 1 or 2  4 (surgery or surgeries or surgical).mp.  5 cataract extraction/  6 cataract*.mp.  7 (orthopedic* or hip or hips or knee*).ti,ab,kw.  8 orthopedic surgery/ or knee surgery/ or hip surgery/  9 or/5-8  10 3 and 4 and 9  11 limit 10 to (english language and yr="2000 -Current")  12 limit 11 to conference abstracts  13 11 not 12 |
| 1974 to 2021 March 25 | Search Update: 2021-03-26  Results: 188 |  |
| 1974 to 2022 October 03 | Search Update: 2022-10-03  Results: 167 |  |
| **EBSCO EconLit** | Searched: 2019-05-14  Results: 14 | **# Query**  S5 S3 AND S4  S4 S1 OR S2  S3 (proprietary or privat* or "for profit" or non-hospital or oursourc* or contract or contracts or contracting or ambulatory)  S2 (orthopedic* or knee or hip* or hips ) AND ( surgery or surgeries or surgical )  S1 cataract* AND (surgery or surgeries or surgical ) |
|  | Search Update:  2021-03-26  Results: 11 | S1 TI ( (private OR privatisation OR privatization OR profit OR non-profit) ) AND TI ( hospital* OR facilit* OR clinic OR clinics OR centre* OR surgery OR surgical OR Health service* OR health care ) AND ( cataract* OR orthopedic* or knee or hip* or hips )  S2 ( (proprietary or privat* or "for profit" or non-hospital or oursourc* or contract or contracts or contracting or ambulatory) ) AND ( surgery OR surgeries OR surgical ) AND ( cataract* OR orthopedic* or knee or hip* or hips )  S3 S1 OR S2 Limiters - Published Date: 20000101-20211231 Search modes - Find all my search terms |
|  | Search Update:  2021-10-03 |  |

| **Grey Literature** | | |
| --- | --- | --- |
| **Google Advanced** | Searched: 2019-05-17  Results: 32 | proprietary OR private OR privately OR for profit OR non hospital OR outsource OR outsourcing OR contract OR contracts OR contracting OR ambulatory cataract OR orthopedic OR knee OR hip filetype:pdf |
|  | Search Update:  2021-04-06  Results: 214 | Search above limited to May 1, 2019 – Apr. 6, 2021 |
|  | Search Update:  2022-10-04 | Search above limited to April 6, 2021 – October 4. 2022 |
| [The King’s Fund](https://www.kingsfund.org.uk) | Searched: 2019-05-21  Results: 2 | Using Google Advanced:  proprietary OR private OR privately OR for profit OR non hospital OR outsource OR outsourcing OR contract OR contracts OR contracting OR ambulatory cataract OR orthopedic OR knee OR hip site:https://www.kingsfund.org.uk/ filetype:pdf |
|  | Search Update:  2021-04-06  Results: 6 | proprietary OR private OR privately OR for profit OR non hospital OR outsource OR outsourcing OR contract OR contracts OR contracting OR ambulatory cataract OR orthopedic OR knee OR hip site:https://www.kingsfund.org.uk/  May 1, 2019 – Apr. 6, 2021 |
|  | Search Update:  2022-10-04  Results: 0 | Search above limited to April 6, 2021 – October 4. 2022 |
| [OECD](https://www.oecd.org) | Searched: 2019-05-21  Results: 2 | Using Google Advanced:  proprietary OR private OR privately OR for profit OR non hospital OR outsource OR outsourcing OR contract OR contracts OR contracting OR ambulatory cataract OR orthopedic OR knee OR hip site:https://www.oecd.org/ filetype:pdf |
|  | Search Update:  2021-04-06  Results: 19 | Search above limited to May 1, 2019 – Apr. 6, 2021 |
|  | Search Update:  2022-10-04  Results: 19 | Search above limited to April 6, 2021 – October 4. 2022 |
| [European Observatory](http://www.euro.who.int/en/about-us/partners/observatory) | Searched: 2019-05-21  Results: 0 | Using Google advanced:  proprietary OR private OR privately OR for profit OR non hospital OR outsource OR outsourcing OR contract OR contracts OR contracting OR ambulatory cataract OR orthopedic OR knee OR hip site:http://www.euro.who.int/en/about-us/partners/observatory |
|  | Search Update:  2021-04-06  Results: 6 | proprietary OR private OR privately OR for profit OR non hospital OR outsource OR outsourcing OR contract OR contracts OR contracting OR ambulatory cataract OR orthopedic OR knee OR hip site:https://eurohealthobservatory.who.int/ |
|  | Search Update:  2022-10-04  Results: 0 | Search above limited to April 6, 2021 – October 4. 2022 |
| INAHTA HTA Database  <https://database.inahta.org/> | Searched: 2022-10-05  Results: 1 | (proprietary OR private OR privately OR "for profit" OR outsource OR outsourcing OR contract*) AND (cataract* OR orthopedic OR knee* OR hip* ) FROM 2000 TO 2022 |
| CADTH  <https://www.cadth.ca/> | Searched: 2022-10-05  Results: 0 | cataract* OR orthopedic OR knee* OR hip* |

| [Commonwealth Fund](https://www.commonwealthfund.org) | Searched: 2019-05-21  Results: 0 | Using Google advanced:  proprietary OR private OR privately OR for profit OR non hospital OR outsource OR outsourcing OR contract OR contracts OR contracting OR ambulatory cataract OR orthopedic OR knee OR hip site:https://www.commonwealthfund.org/ filetype:pdf |
| --- | --- | --- |
|  | Search Update:  2021-04-06  Results: 19 | proprietary OR private OR privately OR for profit OR non hospital OR outsource OR outsourcing OR contract OR contracts OR contracting OR ambulatory cataract OR orthopedic OR knee OR hip site:https://www.commonwealthfund.org/  May 1, 2019 – Apr. 6, 2021 |
|  | Search Update:  2022-10-05  Results: 0 | Search above limited to April 6, 2021 – October 4. 2022 |
| [Conference Board of Canada](https://www.conferenceboard.ca/) | Searched: 2019-05-21  Results: 0  Search Update:  2021-04-06  Results: 11 | Using Google advanced:  proprietary OR private OR privately OR for profit OR non hospital OR outsource OR outsourcing OR contract OR contracts OR contracting OR ambulatory cataract OR orthopedic OR knee OR hip site:https://www.conferenceboard.ca/ |
|  | Search Update:  2021-10-05  Results: 0 | Search above limited to April 6, 2021 – October 4. 2022 |
| [Fraser Institute](https://www.fraserinstitute.org/) | Searched: 2019-05-21  Results: 0 | Using Google advanced:  proprietary OR private OR privately OR for profit OR non hospital OR outsource OR outsourcing OR contract OR contracts OR contracting OR ambulatory cataract OR orthopedic OR knee OR hip site:https://www.fraserinstitute.org/ filetype:pdf |
|  | Search Update:  2021-04-06  Results: 8 | Search above limited to May 1, 2019 – Apr. 6, 2021 |
|  | Search Update:  2021-10-06  Results: 0 | Search above limited to May 1, 2019 – Apr. 6, 2021 |
