# Supplementary material for: A systematic review of the accessibility, acceptability, safety, efficiency, clinical effectiveness, and cost-effectiveness of private cataract and orthopedic surgery clinics
Source: Int J Technol Assess Health Care. 2023 Aug 1;39(1):e47. doi: 10.1017/S0266462323000120 (PMC11570012; doi:10.1017/S0266462323000120)
Supplement: Supplementary file 1 [file S0266462323000120sup.zip › S0266462323000120sup002.docx]

## Additional File 2: Details of the included studies

| **Study** | **Objective** | **Setting** | **Population** | **Conclusion** |
| --- | --- | --- | --- | --- |
| Adie et al. 2012 | To compare outcomes up to one year post knee and hip replacement between patients treated in the public and private sectors | Public compared with private facilities | Scheduled hip or knee surgery patients in Sydney, Australia from April 2007 to December 2008. (331 total, 184 public, 147 private; 215 knees, 116 hips). | Joint replacement outcomes are similar for patients treated in public and private hospitals. Surgeons should manage patient expectations prior to surgery, particularly in private patients. |
| Andersen and Jakobsen 2011 | To investigate how the ownership of hip clinics affects the provision of publicly funded professionalized service behavior, treatment quality and patient satisfaction in a competitive environment | Public compared with private facilities | Hip Arthroplasty patients. 8 149 patients from 36 public and 20 private clinics in Denmark for fiscal years 2007 to 2008. | More research must be done in varying institutional contexts before we can draw firm conclusions. |
| Bannister et al. 2010 | To compare the short-term complications after total hip replacement and total knee replacement at one NHS hospital and two ISTCs | Public compared with private facilities | Hip and knee arthroplasty patients in the UK from October 2003 to March 2005. Patients who had 880 total hip and 874 total knee arthroplasties at a regional orthopedic hospital with 368 total hip and 365 total knee arthroplasties from an NHS and 67 total hip and 86 total knee arthroplasties from a private hospital independent treatment centre. | Results and audits from ISTCs are variable and patients should be warned of this before undergoing treatment at them. |
| Barbieri et al. 2007 | To detect geographic variations in the hospitalization of patients with cataract in Austria and to explain possible differences in demand for and supply of cataract treatment | Public compared with private facilities | Patients admitted to hospital who were diagnosed with cataracts or underwent cataract surgeries in Austria, from 2001-2002. Public hospitals in 2001 (48 443 patients) and of public (47 797 patients) and private hospitals (5100 patients) in 2002. | Differences in the supply by hospitals between districts depend on the availability of hospitals with departments of ophthalmology. The overall demand for cataract surgery in Austria finds its proper supply in many Austrian regions, but needs further development. |
| Browne et al. 2008 | To compare the case-mix and patients' reported outcomes of surgery in ISTCs and in NHS providers | Public compared with private facilities | 769 patients (inguinal hernia, varicose vein, and cataract) treated in six private and 1895 treated in 20 public facilities in England during 2006-07. | The case-mix of patients treated in ISTCs differs from that in NHS providers, in line with the intention of the contracts. Caution is needed in interpreting the observation that patients treated in ISTCs reported slightly better outcomes as very few ISTCs participated, case-mix adjustment might have been insufficient, and patients' reports might have been biased as they were more likely to be satisfied with the way they were treated. |
| Chhabra et al. 2022 | To investigate factors associated with unplanned 30-day readmissions following total knee arthroplasty (TKA), including association with post-hospital  syndrome, patient enablement and transition from hospital to home. | Public compared with private facilities | Consecutive patients over the age of 16 years, attending a 6-week follow-up appointment after undergoing TKA at one of four private and public clinical services between 1 February 2018 and 31 January 2019. | Public patients were significantly more likely to be readmitted within 30 days compared with private patients. The increased likelihood of readmission for public patients might be explained by several contributing factors such as socioeconomic status, longer waiting times resulting in increased impairment and disease complexity. |
| Chard et al. 2011 | To compare characteristics of the patients and outcomes after elective surgery in ISTCs and NHS providers | Public compared with private facilities | Patients undergoing hip or knee replacement (5671 in ISTCs and 14 292 in NHS), inguinal hernia repair (640 and 2023, respectively), or surgery for varicose veins (248 and 1336, respectively) in the UK from June 2008 to September 2009. | Patients undergoing surgery in ISTCs were healthier and had less severe conditions than those undergoing surgery in NHS providers. Some outcomes were better in ISTCs, but differences were small compared with the impact ISTCs could have on the provision of elective services. |
| Cooper et al. 2018 | To evaluate the impact of a government program which facilitated the entry of for- profit ISTCs to compete (the impact of competition from these surgical centres on the efficiency – measured by presurgery length of stay for hip and knee replacement patients – and case mix of incumbent public hospitals) against NHS hospitals in England | Public compared with private facilities | All elective hip and knee replacements (478 226) on patients aged 55-100 performed between financial years 2002/3 and 2008/9 in England. | The entry of ISTCs raised incumbent hospitals productivity. In markets where payments are regulated, competition can raise hospitals efficiency. The entry of ISTCs left public hospitals with a riskier mix of patients. The entry of ISTCs likely to leave existing providers treating a sicker patient mix and worse off financially, unless it is accompanied by a reimbursement system that adequately adjusts payments to reflect patient severity. |
| Fitzpatrick et al. 2004 | To explore sociodemographic and health status factors associated with waiting times both for first outpatient appointment and for total hip replacement surgery | Public compared with private facilities | Patients of recruited physicians in need of hip replacement surgery in England from September 1996 and October 1997. 13 343 surgeries were performed in 143 public hospital sand 390 private firms. | There were significant differences in waiting time according to social, geographical and health care system factors |
| Harris et al. 2019 | To determine the contributing factors of hospital sector (private versus public) variation in revision rates after elective total hip replacement for hip fracture, and elective total knee replacement | Public compared with private facilities | Patients who received primary hip and knee replacement between 2003 and 2016 in Australia. (Hip: 210 828 private, 100 931 public, Knee: 338 259 private, 160 642 public procedures.) | Considerable variation was seen in the revision rate after total hip replacement and total knee replacement between hospital sectors in Australia. The variation was largely due to differences in prosthesis selection. |
| Heath et al. 2022 | To evaluate the hospital- and surgeon-level variation in the severity of patient-reported symptoms prior to THR and TKR surgery. The study also  aimed to examine hospital- and surgeon-level factors associated with symptom severity prior to surgery. | Public compared with private facilities | Patients undergoing primary THR (specifically total conventional hip replacement) or TKR for osteoarthritis between July 2018 and April 2020 who participated in the AOANJRR PROMs  program were included in this analysis (4330 THR patients and 7054 TKR patients) | Patients who underwent primary THR or TKR for osteoarthritis had significantly worse pre-operative symptom severity if their surgery was performed at a public hospital which may reflect variation in access to surgery, and surgeon and patient preferences between these groups. |
| Holom and Hagen 2017 | To compare the quality of care among patients treated at private for-profit hospitals, private non-profit hospitals and public hospitals | Public compared with private facilities | All publicly financed patients having primary total hip (37 897 patients) or primary total knee arthroplasty (25 802 patients) at one of the three hospital types from 2009 to 2014 in Norway. | Quality differences between hospital types were small; however, private non-profit hospitals had significantly lower readmission rates compared with pubic hospitals among patients having total hip arthroplasty. Public hospitals received the larger part of the readmitted patients across hospital types and thus play an essential role in the care of more complex patients and for readmissions, regardless of any quality differences. |
| Karnon et al. 2018 | To evaluate the cost- effectiveness of implementing a private contracting model to meet alternative maximum waiting time targets for publicly funded patients undergoing total knee replacement surgery in Australia | Public compared with private facilities | Non-urgent patients for total knee replacement surgery to a maximum age of 100 years in Australia. Used data inputs from published sources in a modelling study. | The reported cost effectiveness analysis of alternative options for increased government funding of total knee replacement suggests that these options may be more cost-effective than many new pharmaceuticals that are publicly funded in Australia. More evidence on the value of expanded capacity or new models of care may inform new funding models to support such investments and reduced prices for new technologies, leading to more efficient and sustainable publicly funded healthcare systems. |
| Kelly and Stoye 2016 | To identify whether the introduction of independent sector providers led to a shift in demand for elective hip replacement | Public compared with private facilities | Elective hip replacement patients between April 2002 and March 2011 analyzed for 6 781 aggregated geographic areas. | The introduction of independent sector providers increases demand for elective hip replacements remains unchanged. |
| Kelly and Stoye 2020 | To evaluate the impacts of the entry of private hospitals on the publicly funded elective market for hip replacements | Public compared with private providers (including both ISTCs and private hospitals) | Elective hip replacement patients (615,281 patients) between April 2002 and March 2013. | The introduction of private sector increased publicly funded hip replacement capacity but did not improve quality at existing public hospitals. |
| Kirkwood and Pollock 2017 | To analyze in equality of access to elective primary hip arthroplasty by sex, age and socio-economic deprivation by provider type | Public compared with private facilities | Patients receiving publicly funded primary hip arthroplasty (105 872 elective and 48 894 emergency) in Scotland between April 1993 and March 2013. | NHS funding of private sector provision for elective hip arthroplasty was associated with a decrease in public provision and may have contributed to an increase in age and socio-economic inequalities in treatment rates. |
| Koehoorn et al. 2011 | To evaluate the effect of expedited surgical fees paid to physicians in reducing wait time and return to work time | Public compared with private facilities | Workers with accepted workers compensation claim for meniscal injury. 1380 surgeries from 2001 to 2005 in British Columbia, Canada. | The public expedited group had the shortest disability duration from surgical consult to return to work; the expedited fee reduced the surgery wait time (~2 work weeks), and surgeries performed in public hospitals had a shorter return-to-work time (~1 work week). |
| Kruse et al. 2019 | To identify differences between independent treatment centres and general hospitals regarding costs, quality of care, and efficiency | Public (general hospitals) compared with private facilities (independent treatment centres) | 50 000 patients who received cataract care (including academic and tertiary hospitals) from 2013 to 2015. | ISTCs can provide more value for cataract care than general hospitals. |
| Li et al. 2004 | To identify sociodemographic, environmental, and clinical risk factors for the development of postoperative endophthalmitis, using population-based administrative data from Western Australia | Public (metropolitan public, rural public and teaching public hospitals) compared with private facilities | All patients who underwent cataract surgery (117 083 procedures), along with those in whom postoperative endophthalmitis subsequently developed from 1980 to 2000 in Western Australia. | It may be possible to reduce the incidence rate of postoperative endophthalmitis by almost 80% with a systematic approach to the management of elderly patients, length of stay, and clinical protocols. |
| Moscone et al. 2019 | To investigate empirically whether there are differences in healthcare quality between public and private hospitals treating publicly funded patients in Italy, who seek elective and emergency treatments | Public compared with private facilities | Acute myocardial infarction, stroke, hip and knee replacement patients admitted to 189 Italian hospitals located in the Lombardy region between 2012 and 2014. | When public and private hospitals are subject to the same payment system, the evidence on whether private hospitals provide higher quality than public hospitals is mixed. |
| Naylor et al. 2016 | To determine in a knee or hip arthroplasty cohort, if surgery in the private sector predicts greater overall satisfaction with the acute-care experience and greater likelihood to recommend the same hospital | Public compared with private facilities | Hip and knee replacement patients in Australia from 2014-2015. Telephone survey with 457 survey responders (210 private). | Arthroplasty consumers treated in the private sector are not more satisfied with their acute-care experience nor are they more likely to recommend their hospital provider. Rather, avoidance of complications in either sector appears to result in improved satisfaction as well as a greater likelihood that patients would recommend their hospital provider. |
| Pager and McCluskey 2004 | To compare samples of private and public patients’ priorities and satisfaction undergoing day-stay cataract surgery | Public compared with private facilities | Cataract surgery patients. Forty-two public patients and 39 private patients in Sydney, Australia between April and June 2002. | The dearth of information received by public patients raises important questions whether their surgery decision was sufficiently individualized or adequately consented. Patient satisfaction is an important health outcome, and understanding both the domains of satisfaction, as well as their relative importance to patients, is necessary to improve the overall quality of patient care. |
| Perotin et al. 2013 | To investigate whether hospital ownership has an effect on the level of quality reported by patients in the areas of information and interpersonal care, respect for privacy, dignity, hospitality and delays | Public compared with private hospitals | Patients following discharge from public (NHS) and private (ISTC) facilities from 2007 to 2008 in the UK, who are selected to complete a postal questionnaire. 21 680 cases in NHS hospitals and 16 767 cases in ISTCs (linked to 2007 NHS trust Inpatient Survey and the 2007 and 2008 ISTC Inpatient and Day Case quarterly patient surveys) | ISTCs scored better on the all-round quality and prompt discharge factors, but the analysis revealed a pattern in the data in which some hospitals score higher on the series of questions regarding the information provided to patients and relatively lower on questions regarding comfort while others score more highly on questions regarding comfort and relatively less on information questions. Public sector hospitals were closer to the more information end and private sector ones to the comfort one. |
| Siciliani et al. 2013 | To investigate differences in patients’ length of stay between public hospitals, specialized public treatment centres and private treatment centres that provide elective (non- emergency) hip replacement to publicly funded patients | Public hospitals compared to specialized public treatment centres, and private treatment centres | Patients who received primary hip replacement funded by the English NHS for financial year 2006-2007. (42 948 patients, of which 1 841 were treated at 173 public treatment centres and 6 specialized treatment centres, and 938 by 14 private treatment centres). | The specialized public treatment centres and private treatment centres have, on average, respectively 18% and 40% shorter length of stay compared with NHS public hospitals, even after controlling for differences in age, gender, number and type of diagnoses, deprivation and regional variation. |
| Solborg- Bjerrum et al. 2015 | To study the epidemiology and mortality in patients who had cataract surgery in public hospitals and private clinics | Public compared with private facilities | Cataract Surgery patients in Denmark between 2004 and 2012. 243 856 patients (411 140 cataract operations). | In general, patients who had cataract surgery in private hospitals/clinics were healthier, had first eye cataract surgery at an increasingly younger age and had a reduced time interval between cataract surgeries in the two eyes compared to patients who had cataract surgery in public hospitals. The lack of registration of cataract surgery by the private hospitals/clinics limits the quality of the registries. |
| Solborg- Bjerrum et al. 2013 | To compare the risk of postoperative endophthalmitis after cataract surgery at eye departments in public hospitals | Public compared with private facilities | PE cases in cataract surgery patients identified via the National Public Registry in Denmark between 2002 and 2010 (107 701 registered operations). | A few private hospitals/eye clinics had a statistically significantly higher postoperative endophthalmitis risk than the postoperative endophthalmitis risk at the eye departments in public hospitals. The lack of registration with the National Public Registry by the private hospitals/eye clinics suggests that better monitoring of cataract surgery is needed in Denmark to reduce risk and to improve quality. |
| Street et al. 2010 | To assess the characteristics of patients treated in treatment centres and hospitals using the hospital episode statistics (HES) for 2006/7 | Hospitals compared with private treatment centres | Patients receiving care for one of several defined healthcare resource groups (including hip and knee replacements) in the UK in financial year 2006/07. A total of 3 334 535 patients are included in the analysis, of which 77 358 (2.3%) were treated in treatment centres. | Patients treated in NHS treatment centres tended to have significantly more diagnostic and procedure codes than those treated in private treatment centres. |
| Tulp et al. 2020 | To examine if independent treatment centre care outperforms general hospital care within the regulated competition system in Netherlands, by focusing on differences in healthcare quality and price | Independent treatment centres compared with general hospitals | Patients who had anterior cruciate ligament surgery, cataract surgery, total hip replacement, total knee replacement, and carpal tunnel syndrome surgery in Dutch hospitals and ISTCs in 2017. | ISTCs do not seem to deliver higher quality of care compared to general hospitals. |
| Vanhegan et al. 2015 | To investigate the effect on productivity of operating theatres (individual surgeons) working in the ISTCs compared with those working in the Acute Hospital Trust | Public compared with private facilities | Orthopedic surgery patients whose provider held contracts at both a public facility in 2011 and a private facility in 2012. Events from May-July for both years were included. 66 surgeries in 2011, 32 in 2012. | Implementation of the ISTC was detrimental to departmental efficiency, with <50% of the number of patients being treated and a marked reduction in financial productivity. |

Study size, location, and dates are included in the population column. Acronyms: ISTCs: Independent Sector Treatment Centres, NHS: National Health Service
